# Supplementary material for: Data-driven optimized control of the COVID-19 epidemics
Source: Sci Rep. 2021 Mar 22;11:6525. doi: 10.1038/s41598-021-85496-9 (PMC7985510; doi:10.1038/s41598-021-85496-9)
Supplement: Supplementary file 1 — Supplementary material 1. [file 41598_2021_85496_MOESM1_ESM.pdf]

Supplementary Information for Data-driven Optimized control of the Covid19 Epidemics

Afroza Shirin, Yen Ting Lin, Francesco Sorrentino

February 9, 2021

Supplementary Note 1: Model Scheme

The compartmental model described by Eq. 2 in the main text can be visualized in Figure 1 below.

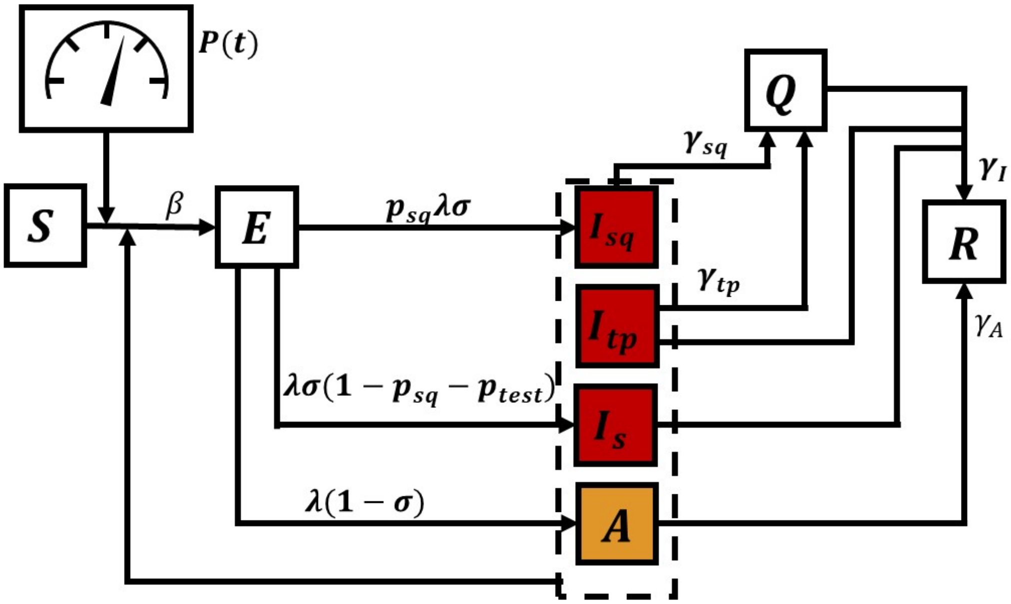

Figure 1: Compartmental model

Supplementary Note 2: Robustness of the optimal control with respect to variations of the parameters c_q and $I_{\max}$

We now investigate the effect of varying the parameters c_q and $I_{\max}$ on the optimal control solutions. Our main result is that we find the optimal control solutions to be robust to variations in both c_q and $I_{\max}$. We choose two values of $I_{\max}$, a value corresponding to the lower value shown in Table 3 of the main manuscript ($\rho = 2/3$) and a value corresponding to the larger value shown in Table 3 of the main manuscript ($\rho = 1$). In Figs. 2 and 3, the states and the optimal controls are plotted for the NYC scenario, for $I_{\max} = 0.0088$ and 0.0132, respectively. In Figs. 4 and 5, the states and the optimal controls are plotted for the LA scenario, for $I_{\max} = 0.0066$ and 0.0097, respectively. In Figs. 6 and 7, the states and the optimal controls are plotted for the Houston scenario, for $I_{\max} =$

0.0088 and 0.0129, respectively. In Figs. 8 and 9, the states and the optimal controls are plotted for the Houston scenario, for $I_{\max} = 0.0046$ and 0.0069, respectively.

All our results in Figs. 2, 3, 4, 5, 6, 7, 8 and 9 show that the optimal control solutions are robust to variations in c_q.

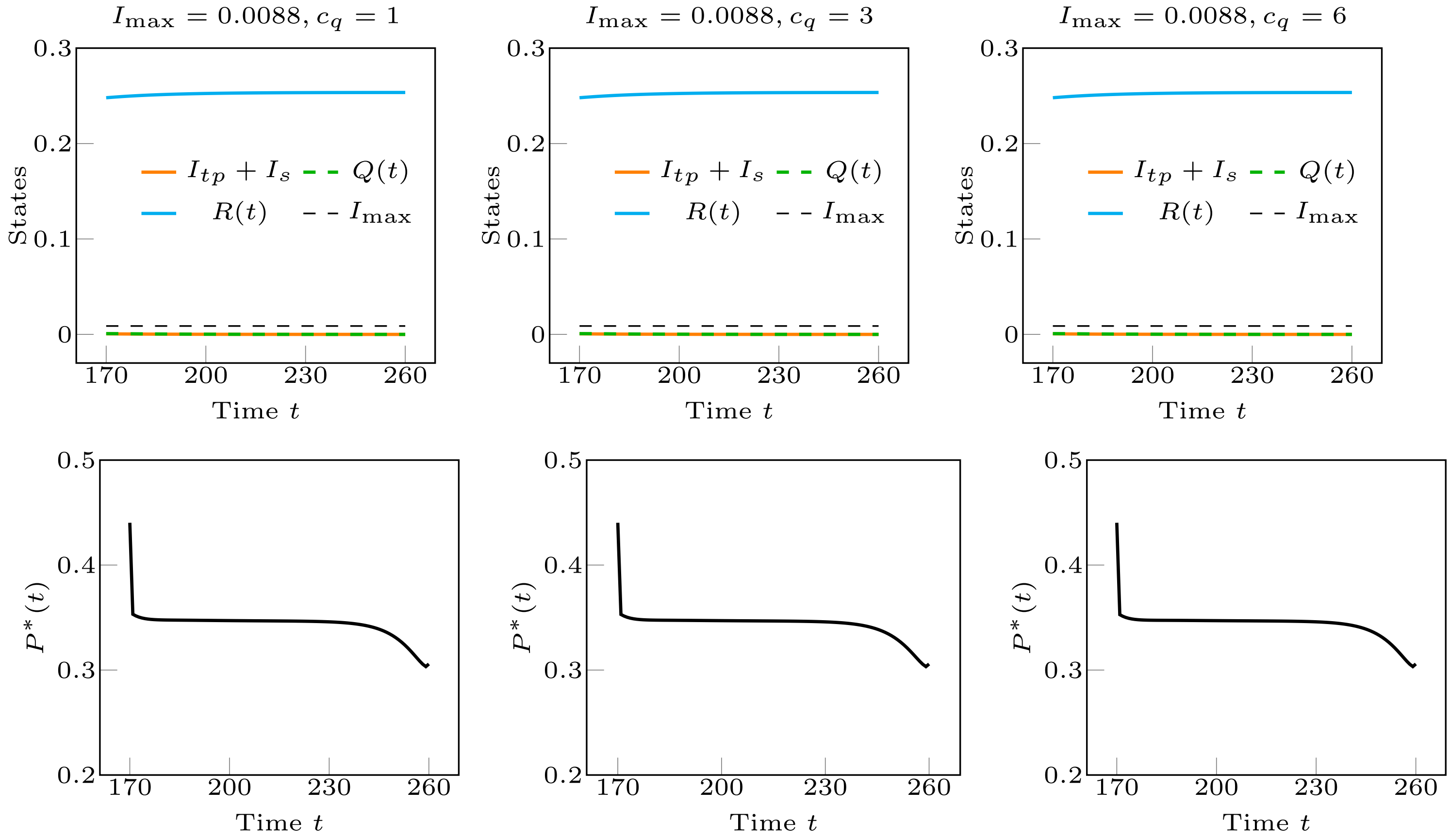

Figure 2: NYC Scenario. $I_{\max} = 0.0088$, minimum of the range.

Figure (10) is analogous to Figure 3 shown in the main manuscript but for the case that $I_{\max}$ are chosen as the maximum values in Table 3 of the main manuscript ($\rho = 1$.) As can be seen the solutions are the same as in Figure 3 of the main manuscript.

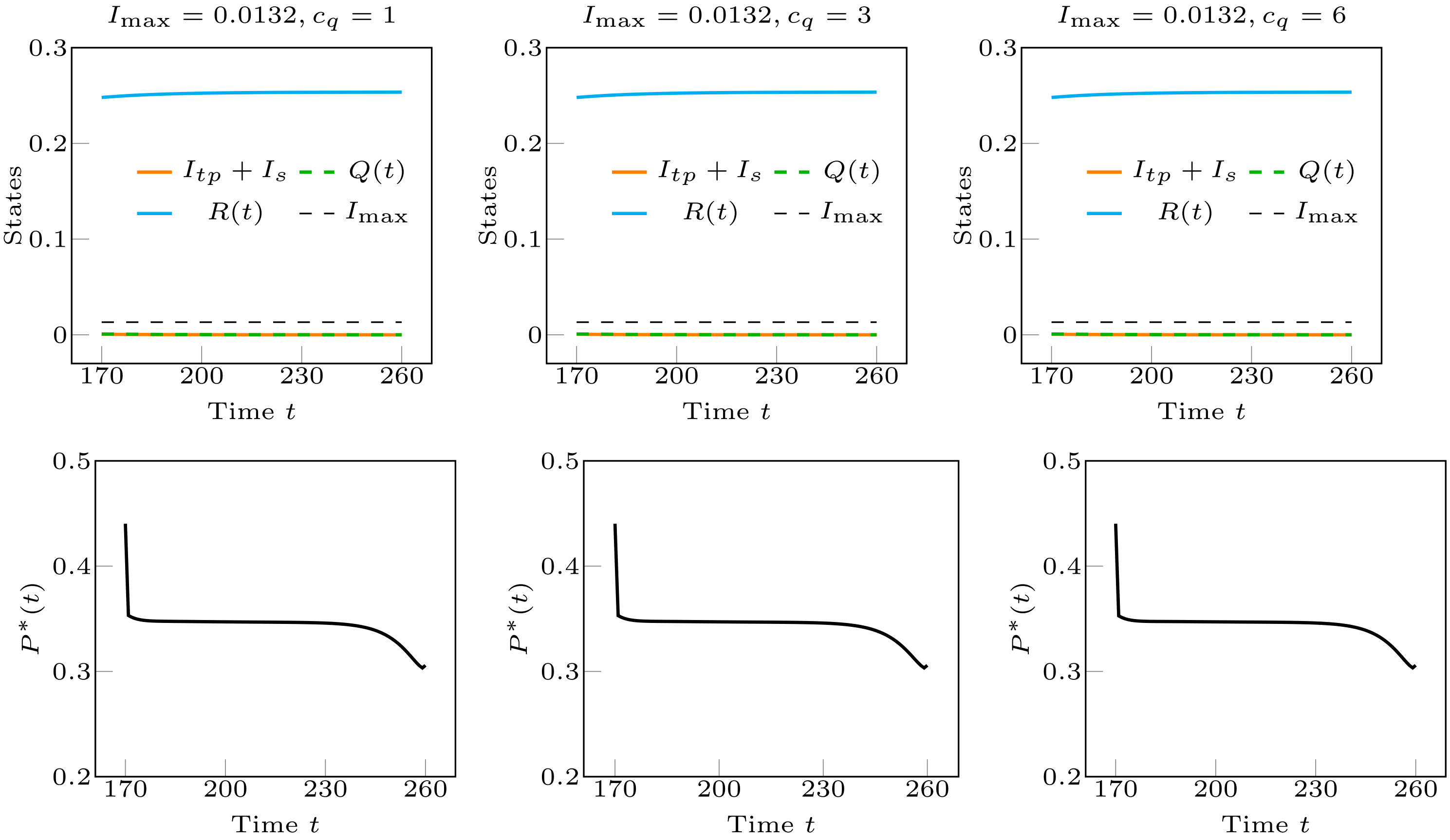

Figure 3: NYC Scenario. $I_{\max} = 0.0132$, maximum of the range.

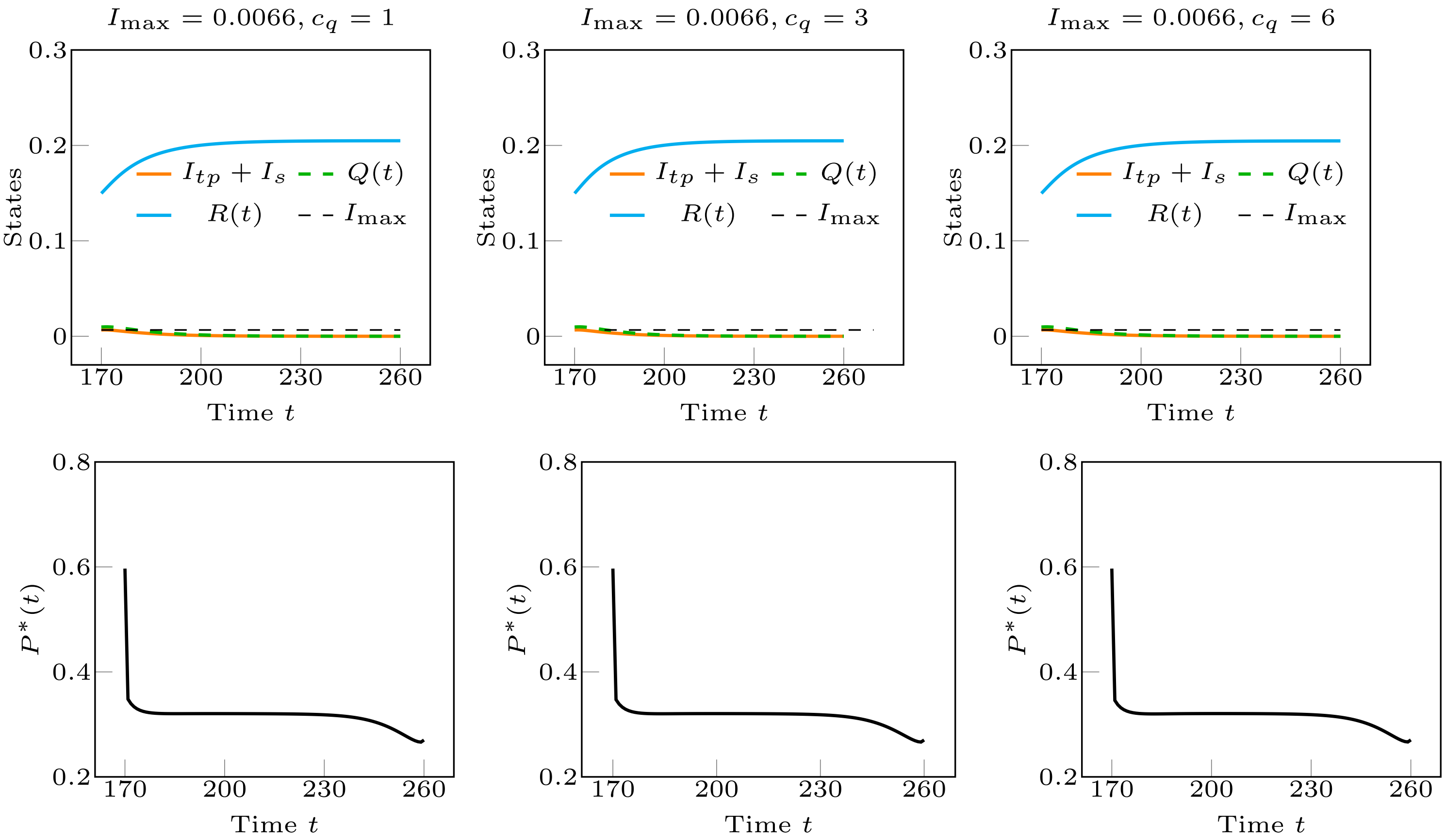

Figure 4: LA Scenario. $I_{\max} = 0.0066$, minimum of the range.

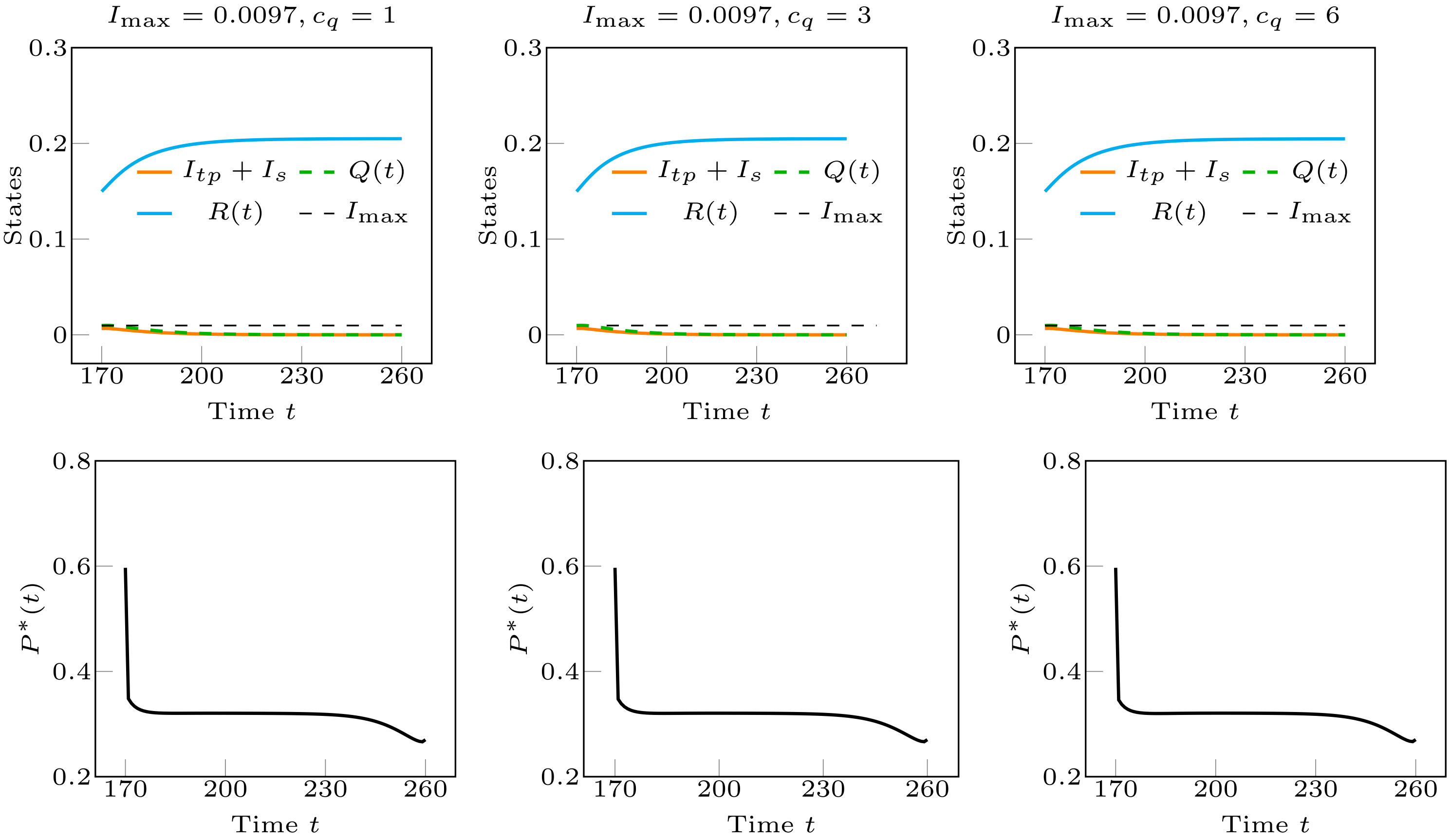

Figure 5: LA Scenario. $I_{\max} = 0.0097$, maximum of the range.

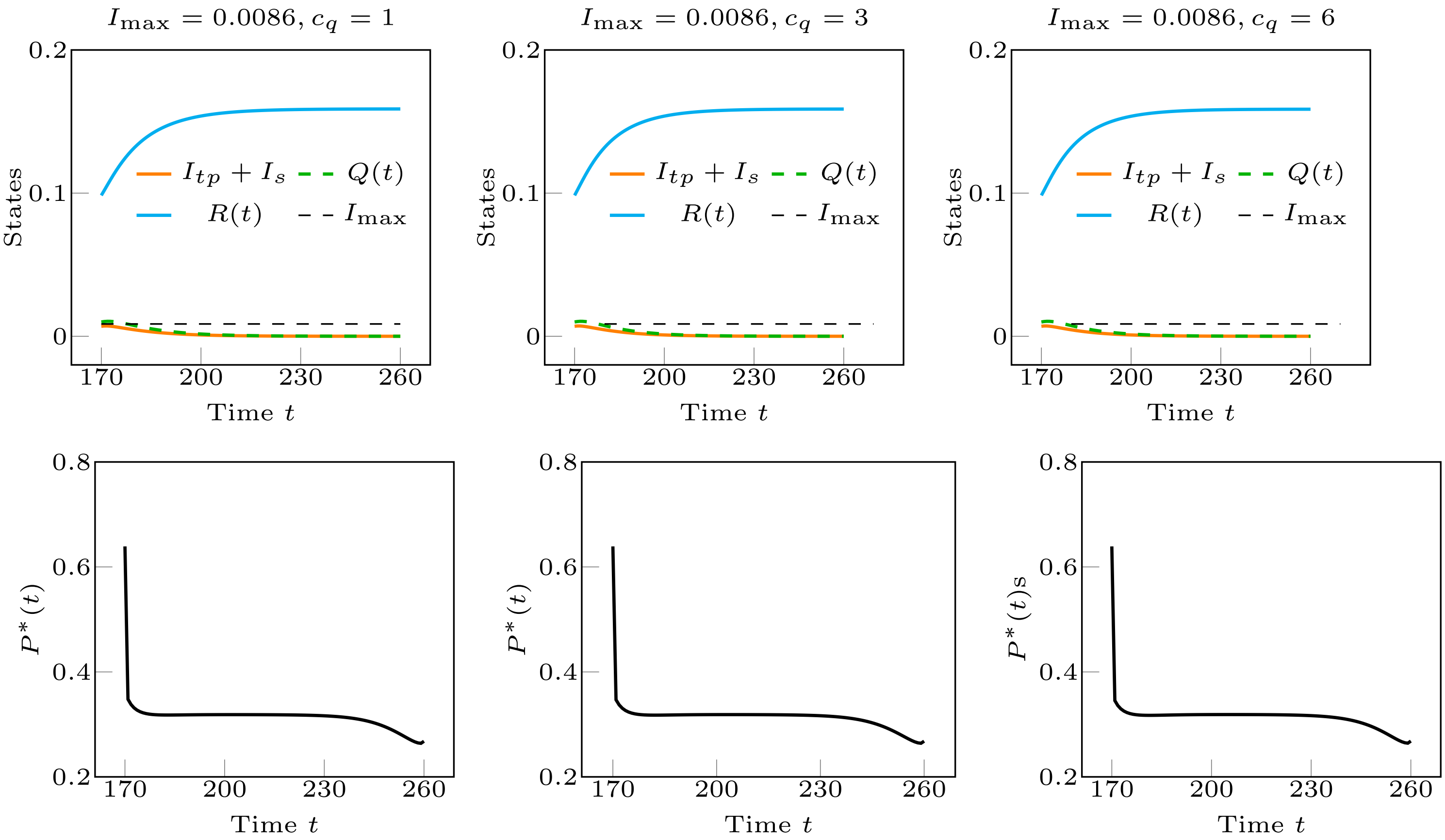

Figure 6: Houston Scenario. $I_{\max} = 0.0086$, minimum of the range.

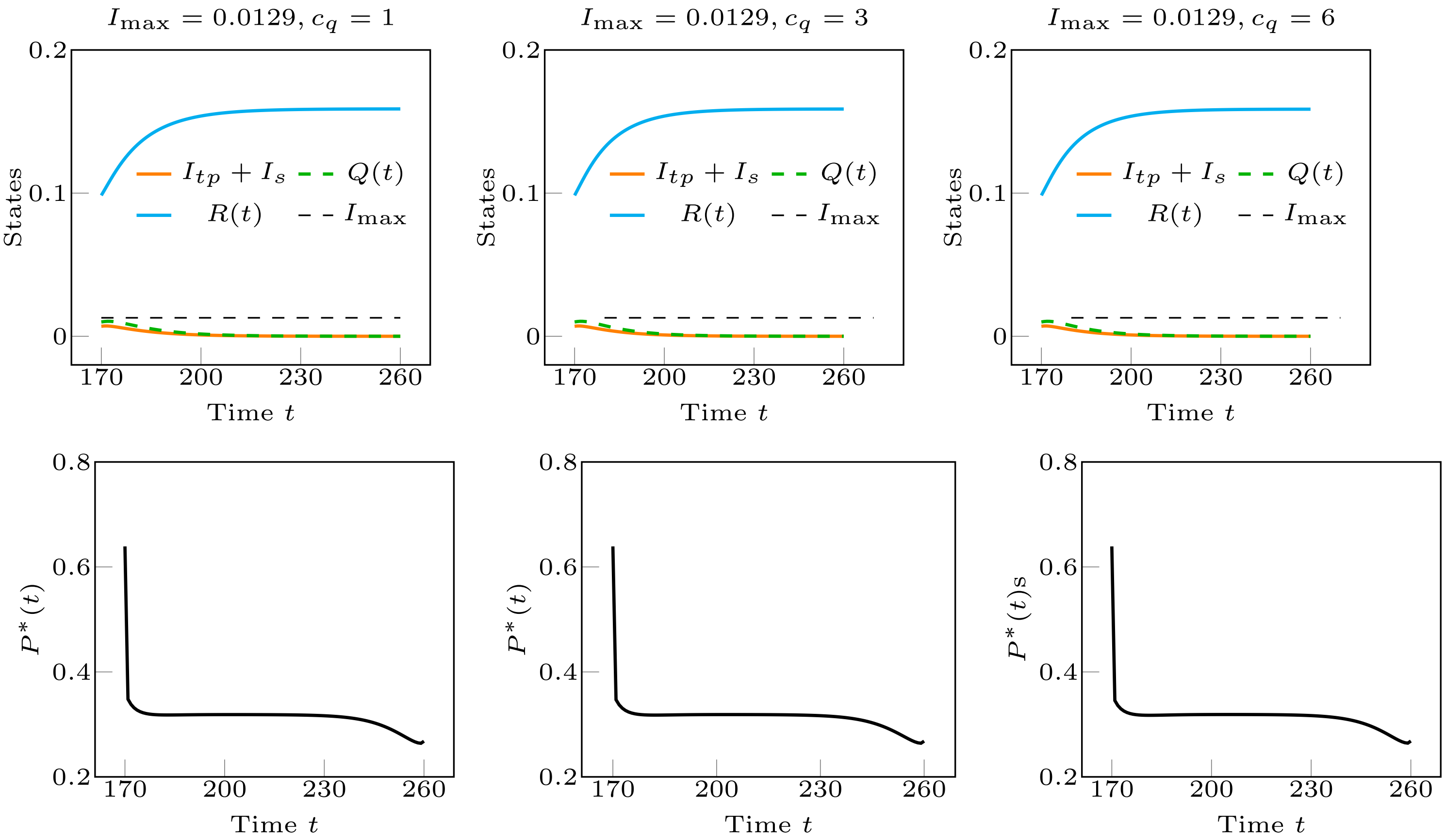

Figure 7: Houston Scenario. $I_{\max} = 0.0129$, maximum of the range.

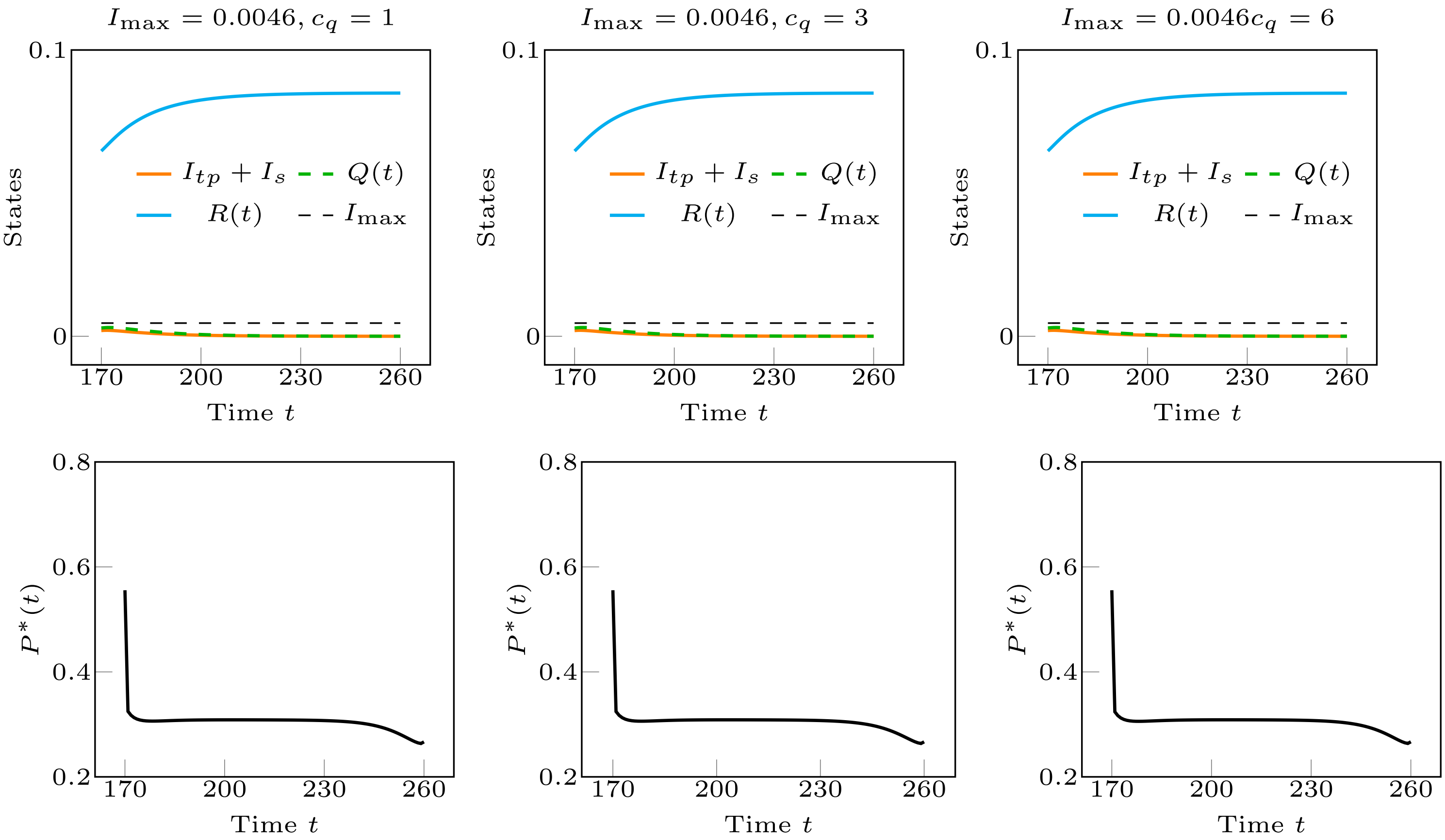

Figure 8: Seattle Scenario. $I_{\max} = 0.0046$, minimum of the range.

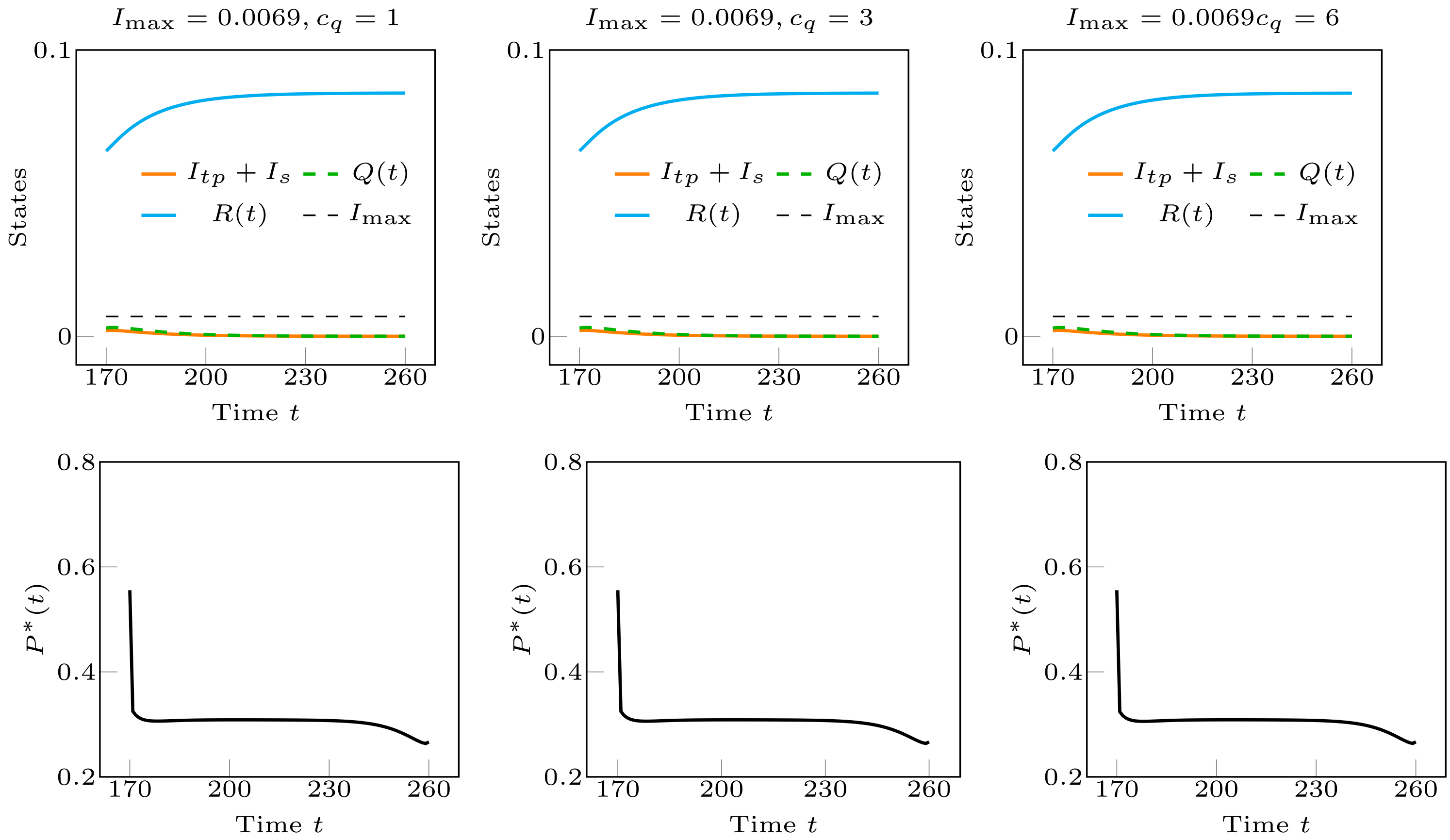

Figure 9: Seattle Scenario. $I_{\max} = 0.0069$, maximum of the range.

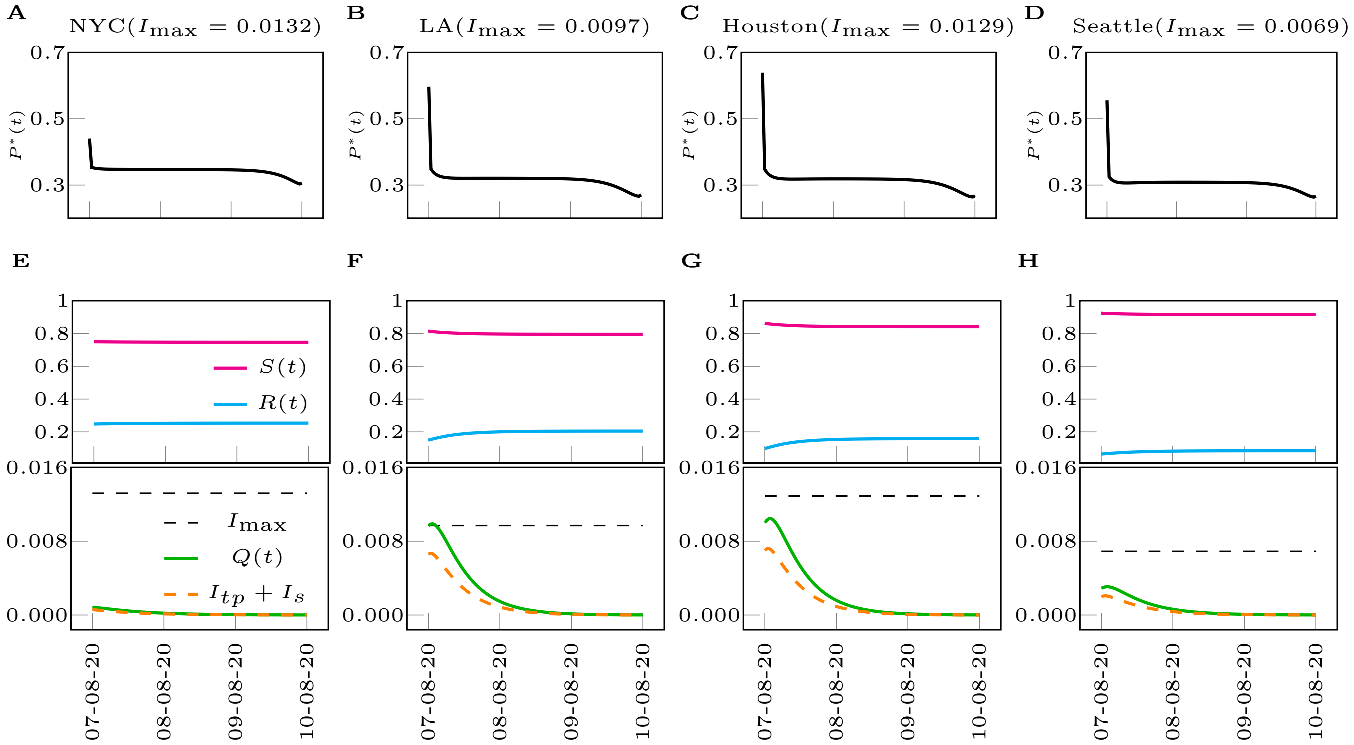

Figure 10: (A-D) Optimal control strategies for the metropolitan cities NYC, LA, Houston, Seattle, respectively. (E-F) Time evolution of the states subject to the optimal control inputs in (A-D). $I_{\max}$ are chosen as the maximum of the range in Table 3 of the main manuscript ($\rho = 1$) and $c_q = 1$. The legends in (F-H) are same as the legend in (E).

Supplementary Note 3: Effects of varying the terminal suppression constraint ϵ

Figures 11, 12, 13, and 14 show the effects of varying the final suppression constraint ϵ on the optimal control solutions, for the cases of the Metropolitan Statistical Areas of NYC, LA, Houston, and Seattle, respectively. For all cities, we see that for large enough ϵ, solutions of type 2 emerge, for which $I_{tp}(t) + I_s(t) = I_{max}$ for certain times t. More specifically, this is seen for $\epsilon = 3.16 \times 10^{-4}$ and $\epsilon = 10^{-3}$ in Figs. 11, 12, and 13 and for $\epsilon = 10^{-3}$ in Fig. 14.

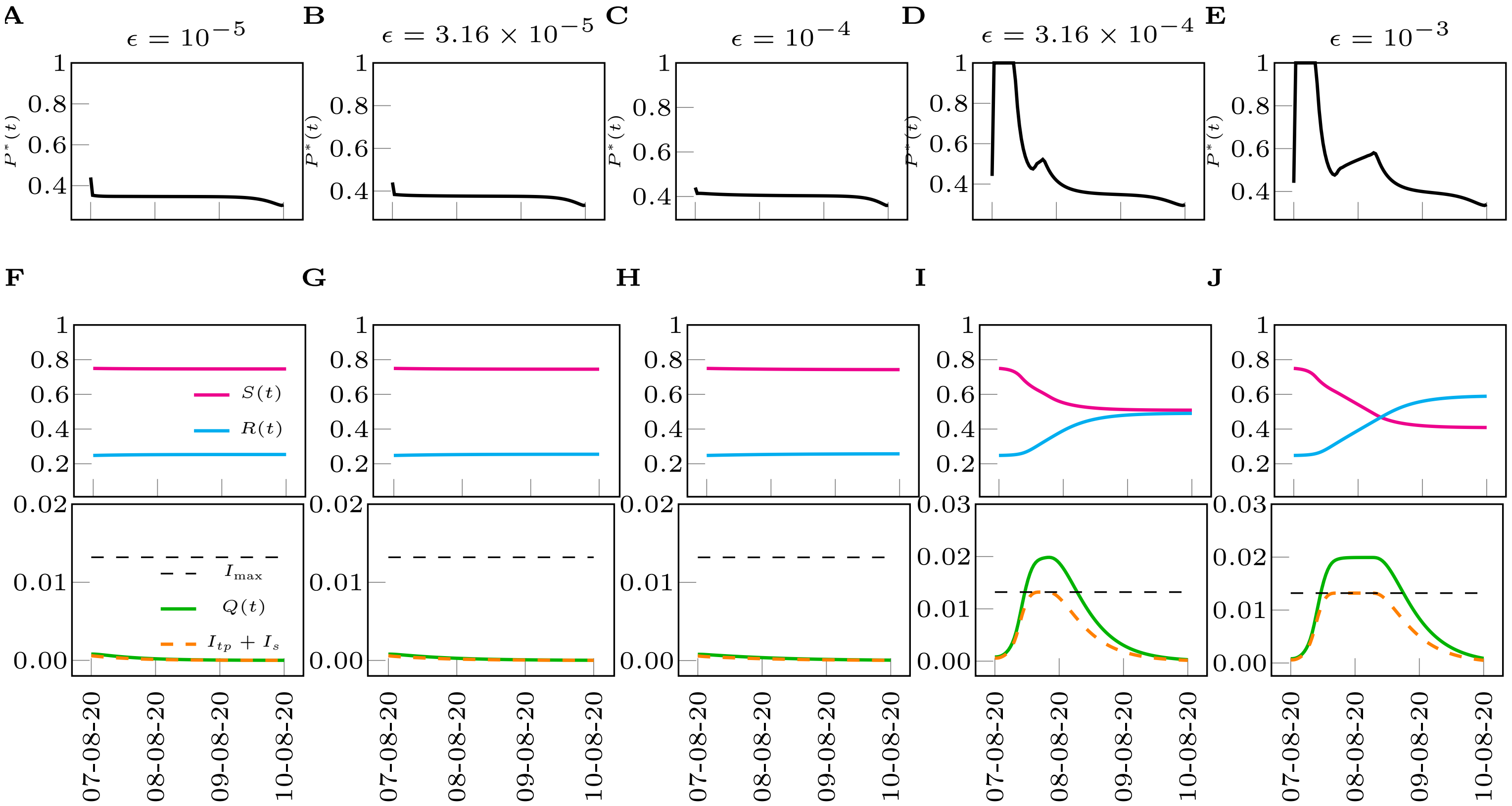

Figure 11: (A-E) Optimal control strategies for the NYC for different values of the parameter ϵ. (F-J) Evolutions of the states subject to the optimal control inputs in (A-E). $I_{\max}$ are chosen from the maximum range of Table 3 of the main manuscript, $\rho = 1$.

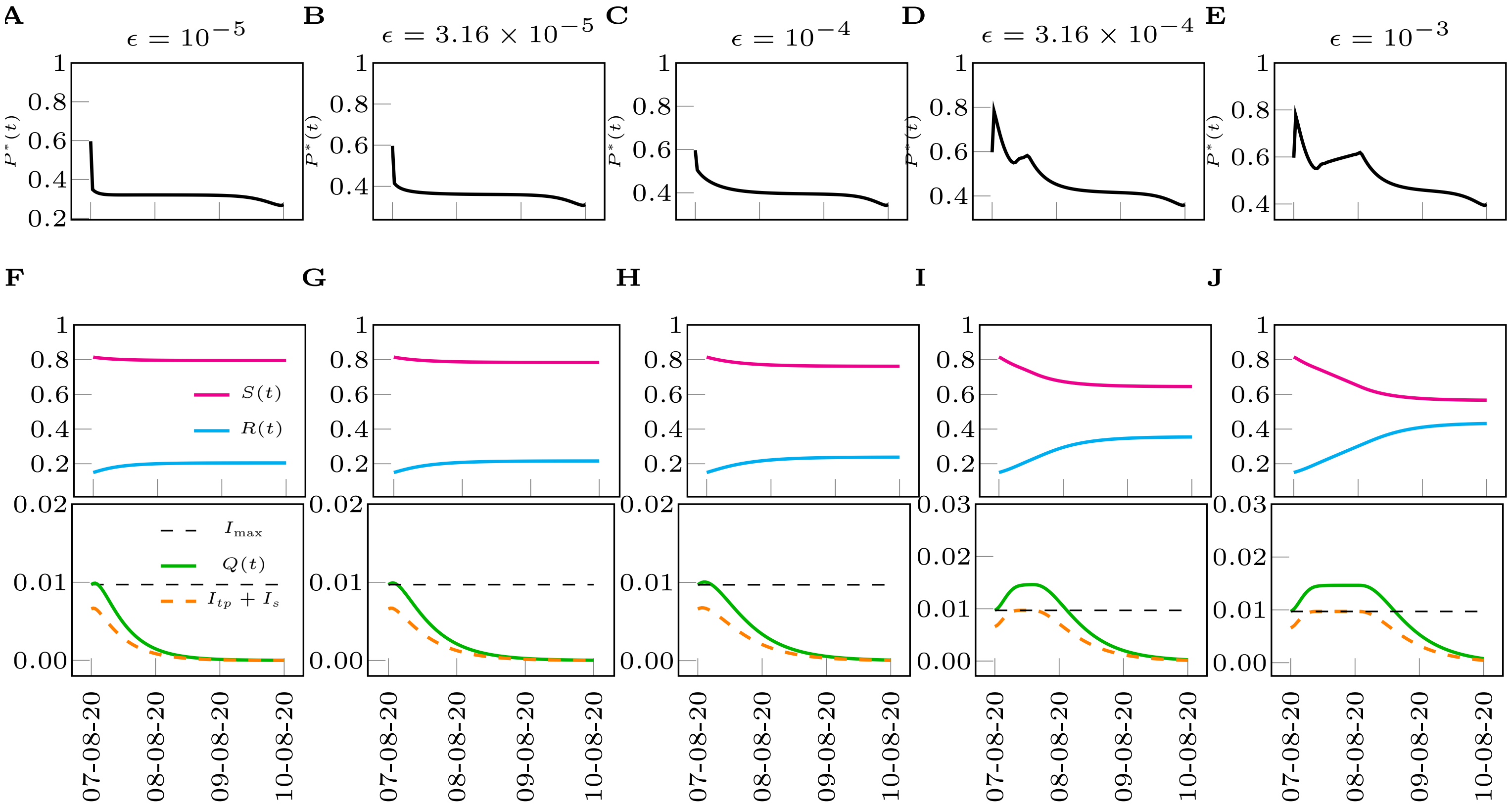

Figure 12: (A-E) Optimal control strategies for LA for different values of the parameter ϵ. (F-J) Evolutions of the states subject to the optimal control inputs in (A-E). $I_{\max}$ are chosen from the maximum range of Table 3 of the main manuscript, $\rho = 1$.

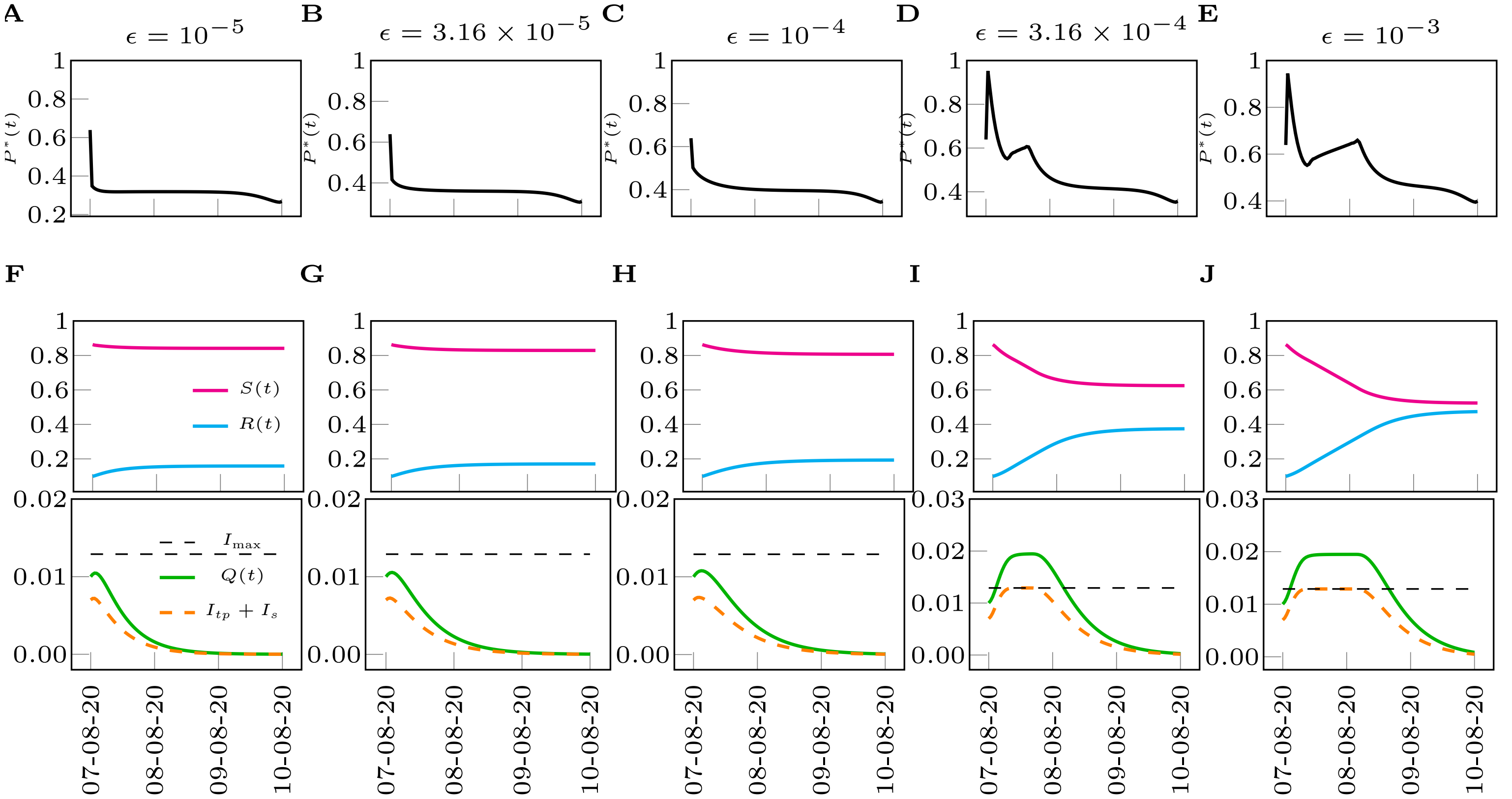

Figure 13: (A-E) Optimal control strategies for Houston for different values of the parameter ϵ. (F-J) Evolutions of the states subject to the optimal control inputs in (A-E). $I_{\max}$ are chosen from the maximum range of Table 3 of the main manuscript, $\rho = 1$.

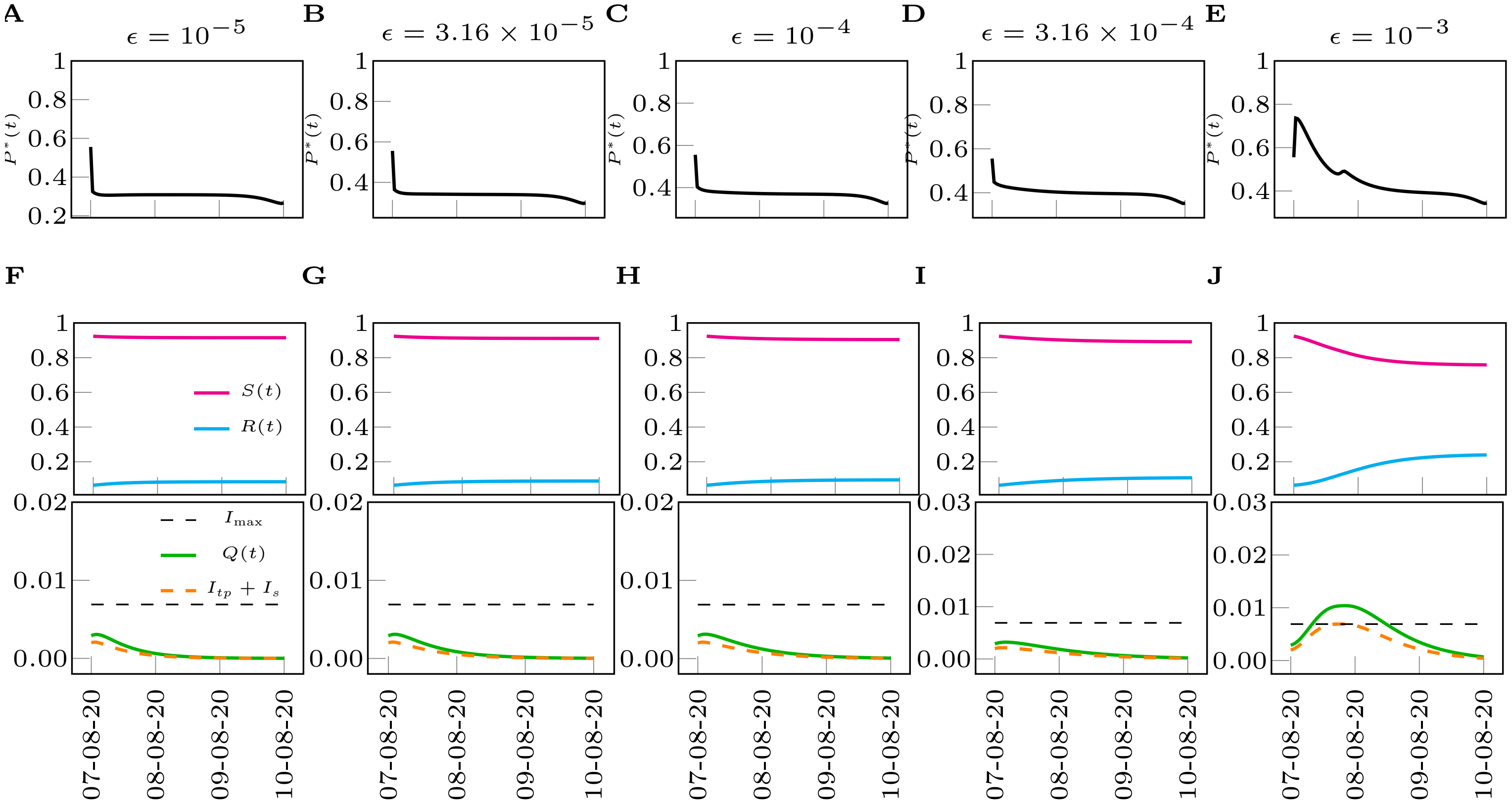

Figure 14: (A-E) Optimal control strategies for Seattle for values of the parameter ϵ. (F-J) Evolutions of the states subject to the optimal control inputs in (A-E). $I_{\max}$ are chosen from the maximum range of Table 3 of the main manuscript, $\rho = 1$.

Supplementary Note 4: Effects of varying t_f

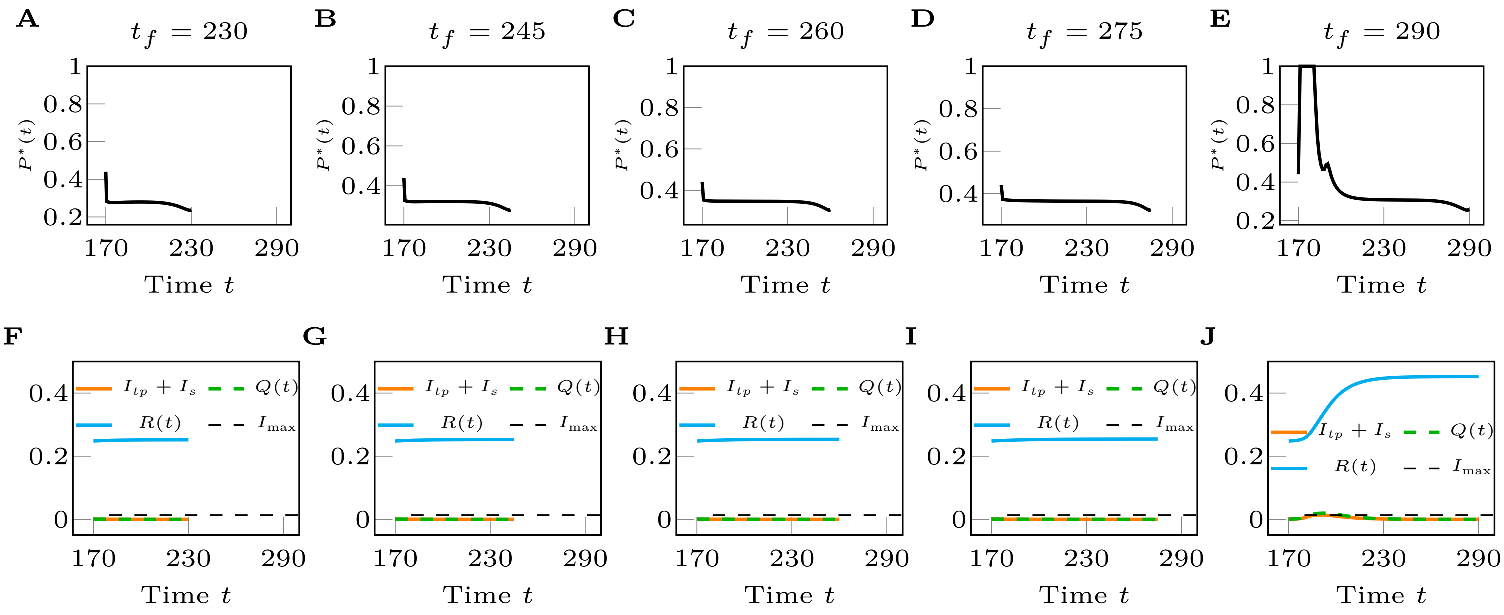

Figure 15: (A-E) Optimal control strategies for the NYC for different final times t_f. (F-J) Evolutions of the states subject to the optimal control inputs. $I_{\max}$ is chosen as the maximum of the range in Table 3 of the main manuscript ($\rho = 1$).

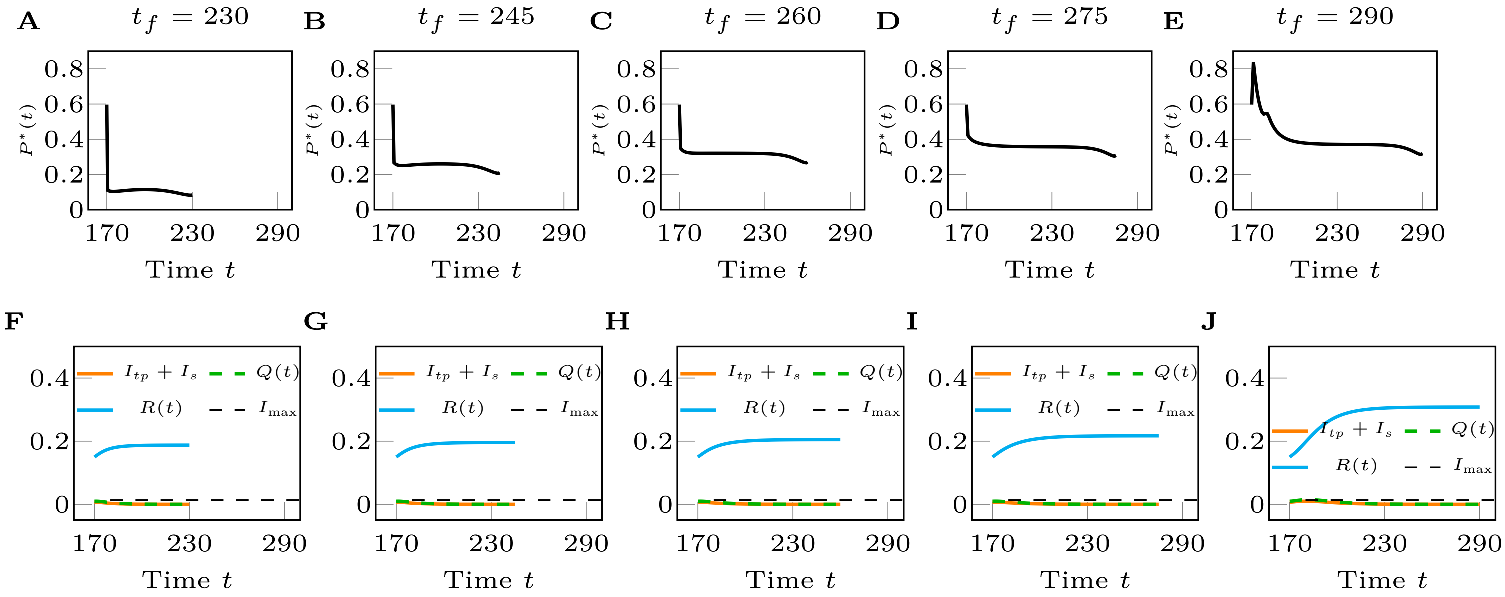

Figure 16: (A-E) Optimal control strategies for LA for different final times t_f. (F-J) Evolutions of the states subject to the optimal control inputs. $I_{\max}$ are chosen as the maximum of the range in Table 3 of the main manuscript ($\rho = 1$) and $\epsilon = 10^{-5}$.

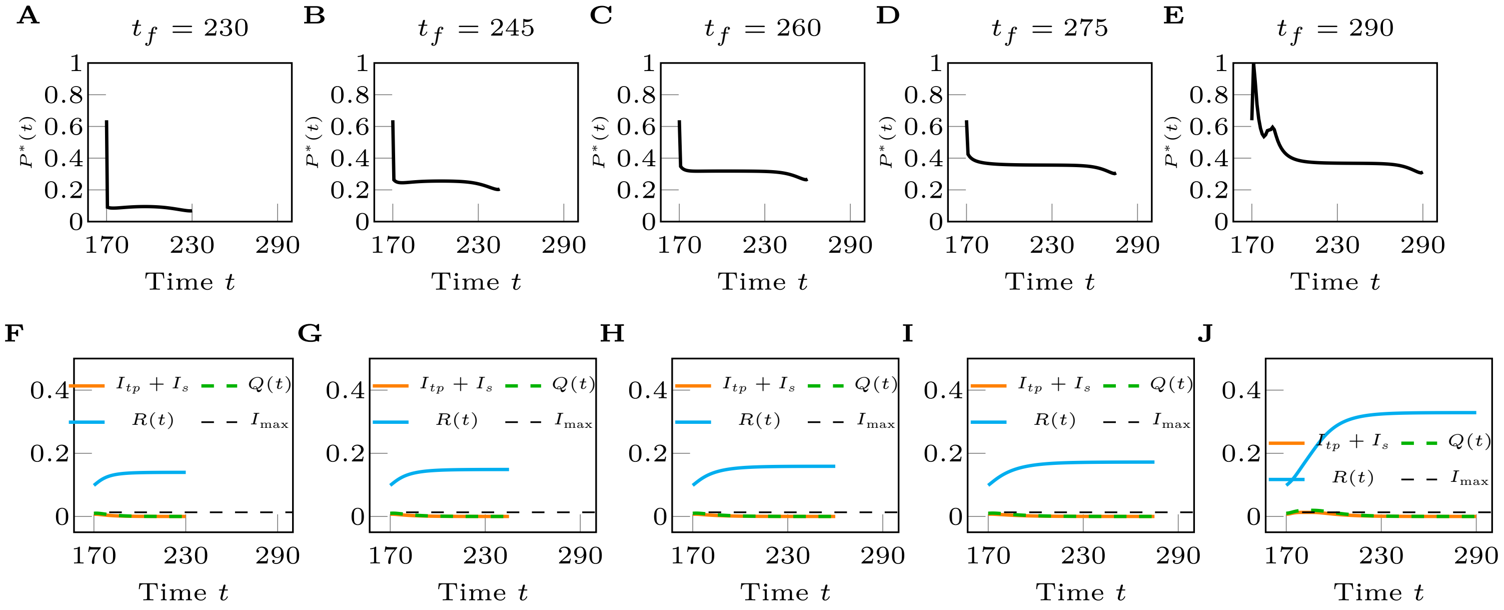

Figure 17: (A-E) Optimal control strategies for Houston for different final times t_f. (F-J) Evolutions of the states subject to the optimal control inputs. $I_{\max}$ are chosen from the maximum range of Table 3 of the main manuscript ($\rho = 1$) and $\epsilon = 10^{-5}$.

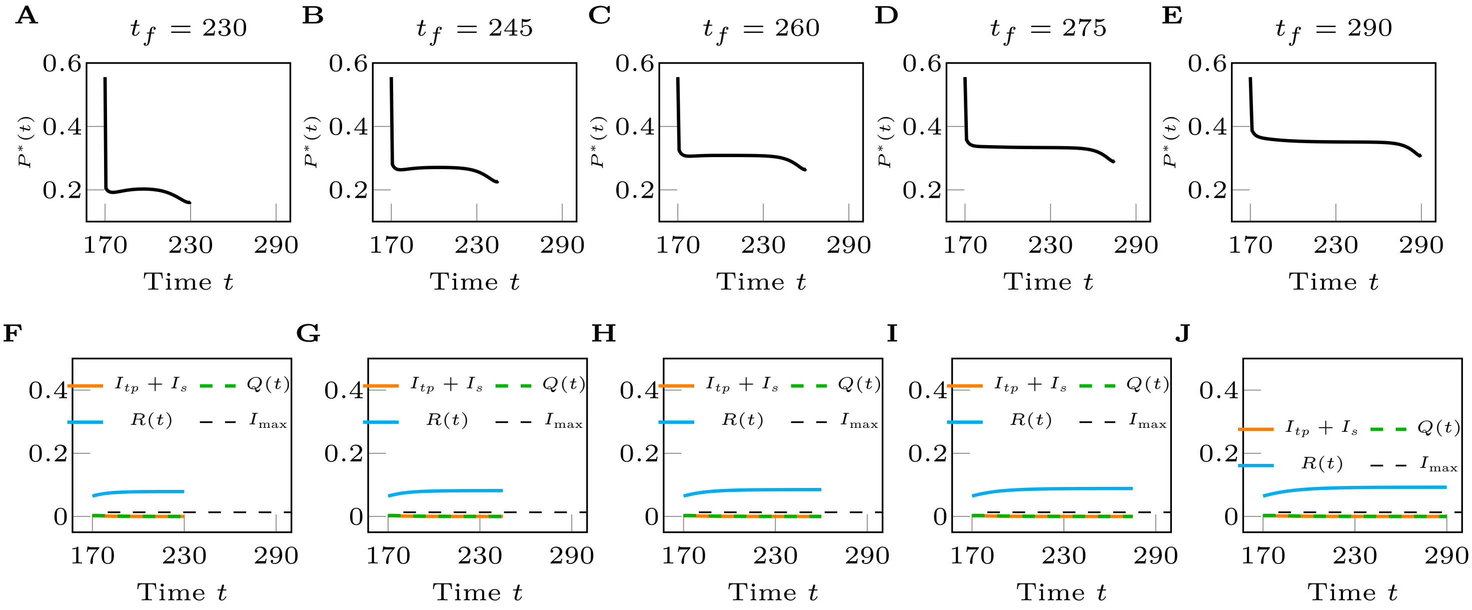

Figure 18: (A-E) Optimal control strategies for Seattle for different final times t_f. (F-J) Evolutions of the states subject to the optimal control inputs. $I_{\max}$ are chosen as the maximum of the range in Table 3 of the main manuscript ($\rho = 1$) and $\epsilon = 10^{-5}$.

Supplementary Note 5: Detailed comparison of different cities

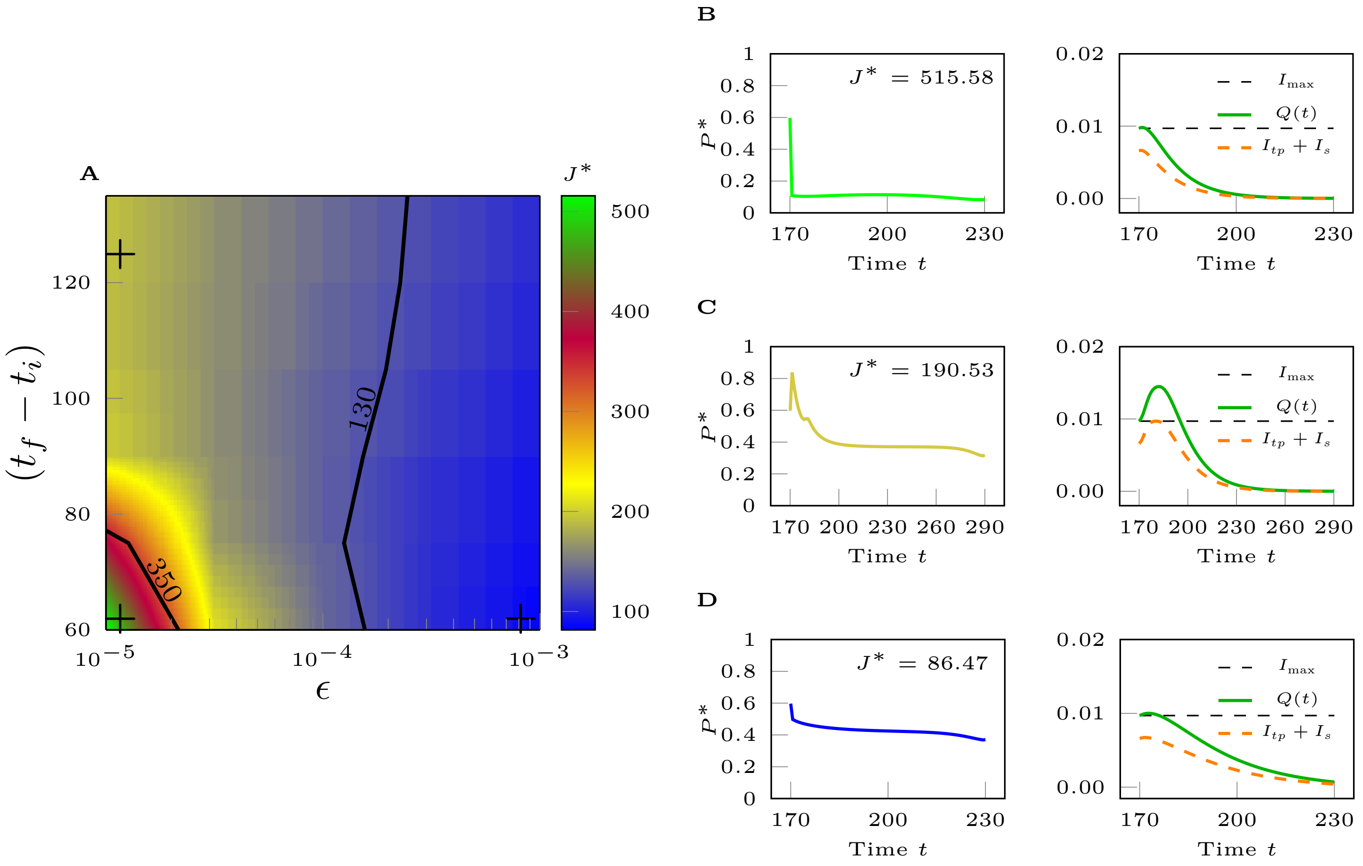

Figure 19: (A) The optimal cost J^* in the $(t_f - t_i)$, ϵ plane. The parameters correspond to the Los Angeles Metropolitan Statistical Area. $I_{\max}$ is chosen as the maximum of the range in Table 3 of the main manuscript . Type 1 solutions (in green) are more expensive than type 2 solutions (in blue.) The regions in yellow/red correspond to the transition between the two types of solutions. (B-D) Time evolutions of the optimal control inputs and states for three different points of the $(t_f - t_i)$-ϵ plane, points shown as plus signs in (A). The parameter c_q and c_p are both set to 1.

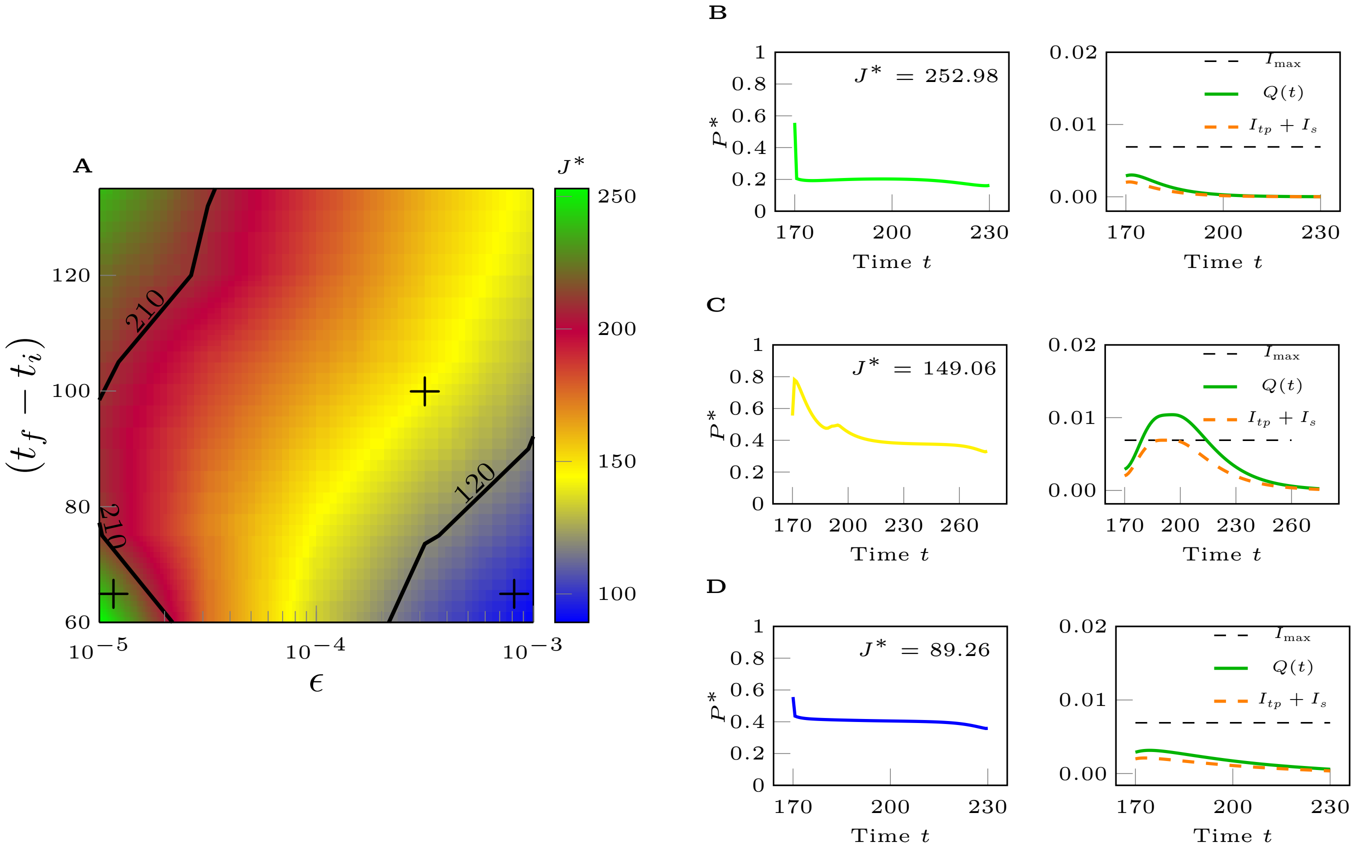

Figure 20: (A) The optimal cost J^* in the $(t_f - t_i)$, ϵ plane. The parameters correspond to the Seattle Metropolitan Statistical Area. $I_{\max}$ is chosen as the maximum of the range in Table 3 of the main manuscript . Type 1 solutions (in red) are more expensive than type 2 solutions (in blue.) The regions in yellow and red correspond to the transition between the two types of solutions. (B-D) Time evolutions of the optimal control inputs and states for three different points of the $(t_f - t_i)$-ϵ plane, points shown as plus signs in (A). The parameter c_q and c_p are both set to 1.

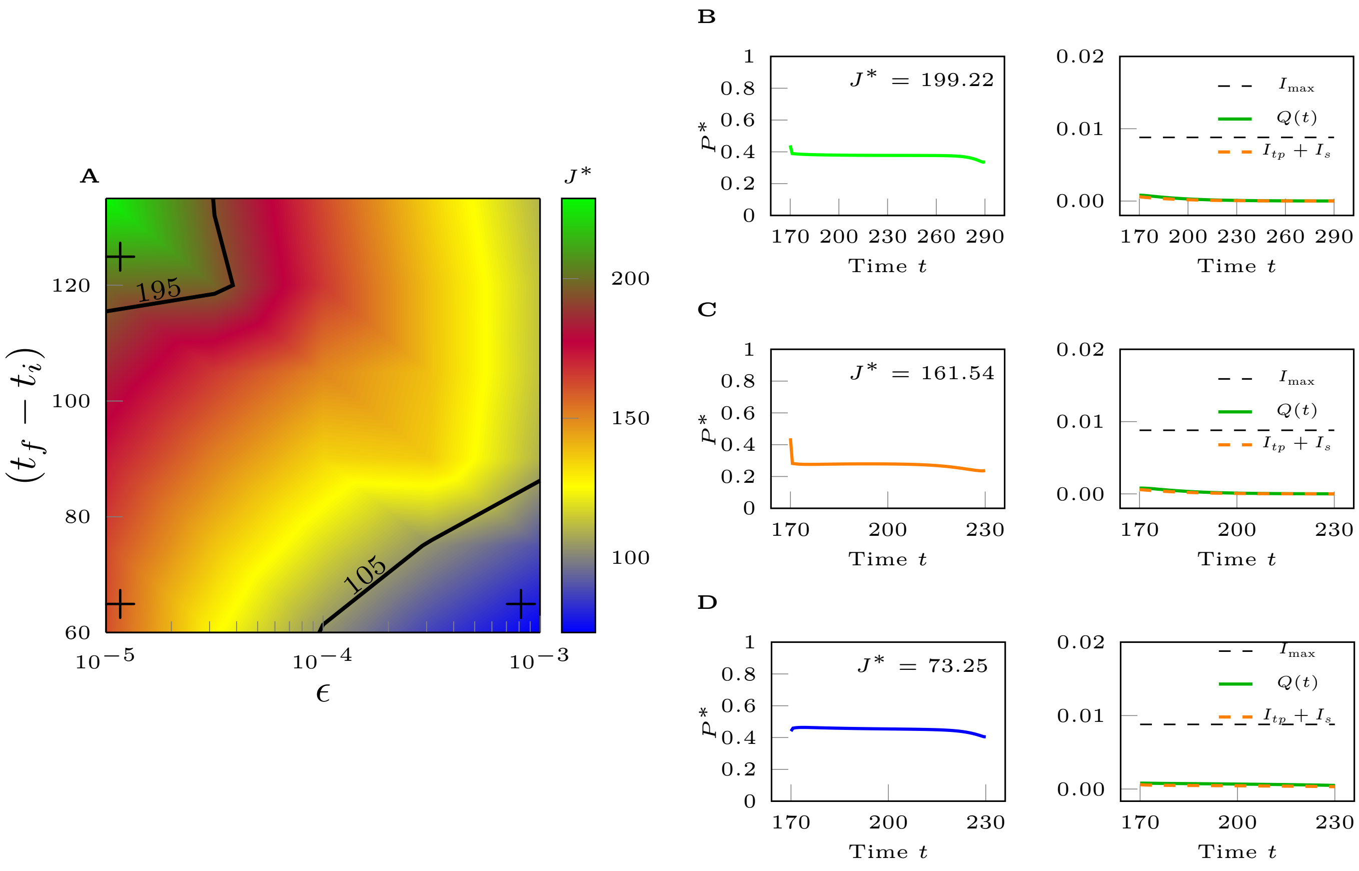

Figure 21: (A) The optimal cost J^* in the $(t_f - t_1)$, ϵ plane. The parameters correspond to the MSA of NYC. $I_{\max}$ is chosen as the maximum of the range in Table 3 of the main manuscript . Type 1 solutions (in red) are more expensive than type 2 solutions (in blue.) The regions in yellow and red correspond to the transition between the two types of solutions. (B-D) Time evolutions of the optimal control inputs and states for three different points of the $(t_f - t_i)$-ϵ plane, points shown as plus signs in (A). The parameters c_p and c_q are both set to 1.

Supplementary Note 6: Herd Immunity Solutions

As stated in the main manuscript, herd immunity solutions arise when the control horizon is very large. Figure 22 shows an example of such solution for the case of NYC, when the final time was set equal to $t_f = 440$. These solutions are characterized by three phases: (I) $\dot{I}(t) > 0$ and $I(t) < I_{\max}$, $t \in [t_i, \tau_1)$, (II) $\dot{I}(t) = 0$ and $I(t) = I_{\max}$, $t \in [\tau_1, \tau_2]$ and (III) $\dot{I}(t) < 0$ and $I(t) < I_{\max}$, $t \in (\tau_2, t_f]$, $t_i \leq \tau_1 \leq \tau_2 \leq t_f$. The second phase has a natural interpretation: one of the objectives is to minimize the usage of social distancing while in the presence of the path constraint $I(t) \leq I_{\max}$, which results in setting $I = I_{\max}$ any time that the number of infected in the absence of controls would exceed $I_{\max}$. From simulations we see that this constant infection state corresponds to approximately setting $\dot{E} = \dot{A} = \dot{Q} = 0$, $t \in [\tau_1, \tau_2]$. Then, the optimal $P^*(t)$ typically has a V-shape (see panel A of Fig. 22), with stricter measures of social distancing only in the central phase.

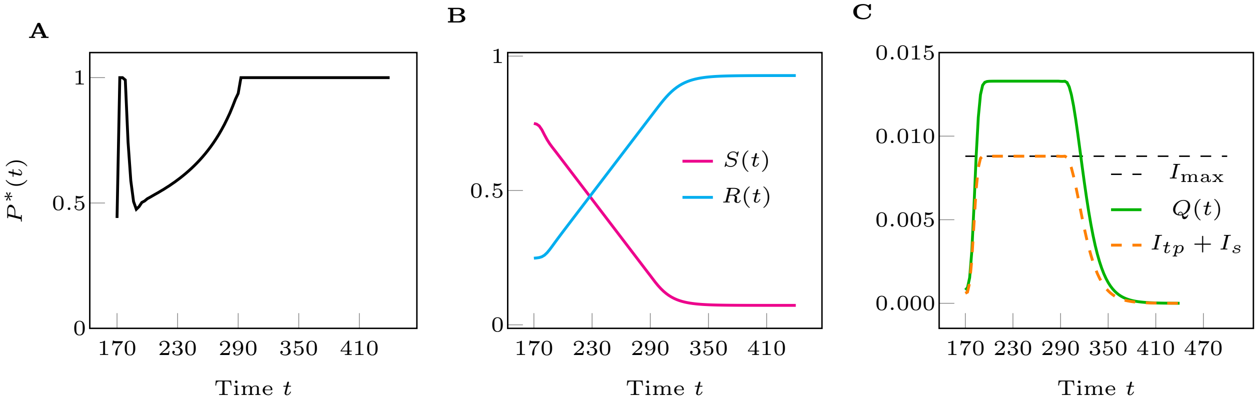

Figure 22: (A) Optimal control strategy for NYC for $t_f = 440$. (B-C) Evaluations of the states to the optimal control input. $I_{\max}$ is chosen as the minimum value in Table 3 of the main manuscript ($\rho = 2/3$) and $\epsilon = 10^{-3}$. The parameters c_p and c_q are both set to 1.

Supplementary Note 7: Implementation of non-optimal control solutions

We have provided an approach to robustly minimize the effects on the economy of social distancing measures in the presence of relevant constraints. However, it is possible that a number of considerations may limit the implementation of such optimized interventions. We are interested in the effects of implementation of non-optimal controls solutions. We thus consider application of a variation of the optimal solution

$$\tilde{P}(t) = \min\{1, (1 + \alpha)P^*(t)\}, \tag{1}$$

Table 1: Violation in the constraints $I_{\max}$ and ϵ for the non-optimal solutions.

NYC: $I_{\max} = 0.0088, c_q = 1, 3, 6$

α	$I_{\max}$	ϵ	J
0	S	S	172.7213
0.1	S	272.4%	148.8441
0.2	S	1.37E+03%	128.9513
0.3	S	5.90E+03%	112.1306
0.4	S	2.39E+04%	97.7420
0.5	S	8.56E+04%	85.3439

LA: $I_{\max} = 0.0066, c_q = 1, 3, 6$

α	$I_{\max}$	ϵ	J
0	S	S	196.7950
0.1	S	13.6355%	170.7582
0.2	S	192.90%	149.0710
0.3	S	652.79%	130.7347
0.4	S	1.82E+03%	115.0389
0.5	S	4.54E+03%	101.4680

Houston: $I_{\max} = 0.0086, c_q = 1, 3, 6$

α	$I_{\max}$	ϵ	J
0	S	S	198.7045
0.1	S	9.17%	172.4965
0.2	S	177.67%	150.6672
0.3	S	602.96%	132.2108
0.4	S	1.67E+03%	116.4129
0.5	S	4.11E+03%	102.7529

Seattle: $I_{\max} = 0.0046, c_q = 1, 3, 6$

α	$I_{\max}$	ϵ	J
0	S	S	206.7624
0.1	S	65.96%	179.8001
0.2	S	432.45%	157.3389
0.3	S	1.65E+03%	138.3460
0.4	S	5.57E+03%	122.0895
0.5	S	1.73E+04%	108.0463

$\alpha > 0$, and analyze violations of the constraints $I_{\max}$ and ϵ as α is varied. Increasing values of α indicate stronger deviations of the control action from the optimal one. Table 1 summarizes application of such non-optimal interventions in all four cities, with α varying from 0.1 to 0.5. The letter 'S' stands for constraint satisfied, otherwise we report the percentage by which the constraint is violated. We see that the constraint on $I_{\max}$ remains always satisfied (this is expected as this constraint is not dominant) but strong violations of the suppression constraint ϵ are otherwise recorded. From Table 1 we see that for $\alpha = 0.5$ the constraint on ϵ in the four areas of interest is violated by an amount that varies from roughly 4000% for Houston to 80000% for NYC (which corresponds to a fraction of infected people at the final time in the order of 10^{-2}.) These results are opposite to those observed previously. The city that is less resilient to variation in the control input is NYC, which was previously reported to have received closer to optimal control interventions. This is due to the higher β for NYC (see Table 1) and to the fact that the optimal $P^*(t)$ for NYC is higher than for the other cities (corresponding to less strict social distancing required); as a result, increasing $P(t)$ further leads to the poorest outcome. This also highlights the risk for resurgences of the epidemics after partial suppression has been achieved [8], which is currently seen in different parts of the world.

Supplementary Note 8: Pseudo-spectral Optimal Control

Pseudo-Spectral Optimal Control (PSOC) is a computational method for solving optimal control problems. PSOC [5, 7] has provided a numerical tool to let scientists and engineers solve optimal control problems

$$\begin{aligned}
\min_{\mathbf{u}(t)} \quad & J(\mathbf{x}(t), \mathbf{u}(t), t) = E\left(\mathbf{x}(t_i), \mathbf{x}(t_f), t_i, t_f\right) + \int_{t_i}^{t_f} F\left(\mathbf{x}(t), \mathbf{u}(t), t\right) dt \\
\text{s.t.} \quad & \dot{\mathbf{x}}(t) = \mathbf{f}(\mathbf{x}(t), \mathbf{u}(t), t) \\
& \mathbf{e}^L \leq \mathbf{e}(\mathbf{x}(t_i), \mathbf{x}(t_f), t_i, t_f) \leq \mathbf{e}^U \\
& \mathbf{h}^L \leq \mathbf{h}(\mathbf{x}(t), \mathbf{u}(t), t) \leq \mathbf{h}^U \\
& t \in [t_i, t_f]
\end{aligned} \tag{2}$$

reliably and efficiently in applications such as guiding autonomous vehicles and maneuvering the international space station [7]. PSOC is an approach by which an OCP can be discretized by approximating the integrals by quadratures and the time-varying states and control inputs with interpolating polynomials. Here we summarize the main concepts behind the PSOC. We choose a set of $N+1$ discrete times $\{\tau_i\}$ $i = 0, 1, \ldots, N$ where $\tau_0 = -1$ and $\tau_N = 1$ with a mapping between $t \in [t_i, t_f]$ and $\tau \in [-1, 1]$. The discretization scheme includes the endpoints and is normalized by the mapping,

$$t = \frac{t_f - t_i}{2}\tau + \frac{t_f + t_i}{2} \tag{3}$$

The times $\{\tau_i\}$ are chosen as the roots of an $(N+1)$th order orthogonal polynomial such as Legendre polynomials or Chebyshev polynomials. The choice of dicretization scheme is important to the convergence of the full discretized problem. For instance, if we choose the roots of a Legendre polynomial as the discretization scheme, the associated quadrature weights can be found in the typical way for Gauss quadrature. The time-varying states and control inputs are found by approximating them with Lagrange interpolating polynomials,

$$\hat{\mathbf{x}}(\tau) = \sum_{i=0}^{N} \hat{\mathbf{x}}_i L_i(\tau) \tag{4a}$$

$$\hat{\mathbf{u}}(\tau) = \sum_{i=0}^{N} \hat{\mathbf{u}}_i L_i(\tau), \tag{4b}$$

where $\hat{\mathbf{x}}(\tau)$ and $\hat{\mathbf{u}}(\tau)$ are the approximations of $\mathbf{x}(\tau)$ and $\mathbf{u}(\tau)$, respectively, and $L_i(\tau)$ is the ith Lagrange interpolating polynomial. The Lagrange interpolating polynomials are defined as,

$$L_i\tau = \prod_{j=0, j\neq i}^{N} \frac{\tau - \tau_j}{\tau_i - \tau_j} \tag{5}$$

The dynamical system is approximated by differentiating the approximation $\hat{\mathbf{x}}(\tau) = \sum_{i=0}^{N} \hat{\mathbf{x}}_i L_i(\tau)$ with respect to time.

$$\frac{d\hat{\mathbf{x}}}{d\tau} = \sum_{i=0}^{N} \hat{\mathbf{x}}_i \frac{dL_i}{d\tau} \tag{6}$$

Let $D_{k,i} = \frac{d}{d\tau} L_i(\tau_k)$ which allows one to rewrite the original dynamical system constraints in (2) as the following set of algebraic constraints.

$$\begin{aligned} \sum_{i=0}^{N} D_{k,i}\hat{\mathbf{x}}_i - \frac{t_f - t_i}{2}\mathbf{f}(\hat{\mathbf{x}}_k, \hat{\mathbf{u}}_k, \tau_k) = \mathbf{0}_n, \quad k = 1, \ldots, N \\ \hat{\mathbf{x}}_N - \hat{\mathbf{x}}_0 - \sum_{k=1}^{N}\sum_{i=0}^{N} w_k D_{k,i}\hat{\mathbf{x}}_i = \mathbf{0}_n \end{aligned} \tag{7}$$

The last set of algebraic constraints arise from the consistency condition $\int_{t_i}^{t_f} \dot{\mathbf{x}}(t)dt = \mathbf{x}(t_f) - \mathbf{x}_0$. Similarly to the consistency condition, the integral in the cost function is,

$$J = \int_{t_i}^{t_f} F(\mathbf{x}, \mathbf{u}, t) \approx \hat{J} = \frac{t_f - t_i}{2} \sum_{k=1}^{N} F(\hat{\mathbf{x}}_k, \hat{\mathbf{u}}_k, \tau_k) \tag{8}$$

The original time-varying states, control inputs, the dynamical equations constrained and the cost function are now discretized approximation of the continuous NLP problem. Thus the discretized approximation of the original OCP is compiled into the following nonlinear programming (NLP) problem.

$$\begin{aligned} \min_{\substack{\mathbf{u}_i \\ i=0,\ldots,N}} \quad & \hat{J} = \frac{t_f - t_i}{2} \sum_{i=0}^{N} w_i f(\hat{\mathbf{x}}_i, \hat{\mathbf{u}}_i, \tau_i) \\ \text{s.t.} \quad & \sum_{i=0}^{N} D_{k,i}\hat{\mathbf{x}}_i - \frac{t_f - t_i}{2}\mathbf{f}(\hat{\mathbf{x}}_k, \hat{\mathbf{u}}_k, \tau_k) = \mathbf{0}, \ k = 0, \ldots, N \\ & \hat{\mathbf{x}}_N - \hat{\mathbf{x}}_0 - \sum_{k=1}^{N}\sum_{i=0}^{N} w_k D_{k,i}\hat{\mathbf{x}}_i = \mathbf{0}_n \\ & \mathbf{e}^L \leq \mathbf{e}(\hat{\mathbf{x}}_0, \hat{\mathbf{x}}_N, \tau_0, \tau_N) \leq \mathbf{e}^U \\ & \mathbf{h}^L \leq \mathbf{h}(\hat{\mathbf{x}}_k, \hat{\mathbf{u}}_k, \tau_k) \leq \mathbf{h}^U, \ k = 0, \ldots, N \\ & t_i = \frac{t_f - t_i}{2}\tau_i + \frac{t_f + t_i}{2} \end{aligned} \tag{9}$$

With the above results, we now present the application to the full multi-phase optimal control problem. In general, let us assume there are $p > 1$ phases where we set $p = 2$ for simplicity. Each phase is active within

the interval $t \in [t_i^{(p)}, t_f^{(p)}]$. In each phase there is a cost function $J^{(p)}$, a dynamical system $\mathbf{f}^{(p)}$, a set of endpoint constraints $\mathbf{e}^{(p)}$, and a set of path constraints $\mathbf{h}^{(p)}$. If two phases, p and q, are linked, then there also exists a set of linkage constraints $\Phi^{(p,q)}$.

$$\begin{aligned}
\min_{\mathbf{u}^{(p)}} \quad & \sum_{p=1}^{P} J^{(p)} = \sum_{p=1}^{P} \int_{t_i^{(p)}}^{t_f^{(p)}} F^{(p)}(\mathbf{x}^{(p)}, \mathbf{u}^{(p)}, t) dt \\
\text{s.t.} \quad & \dot{\mathbf{x}}^{(p)}(t) = \mathbf{f}^{(p)}(\mathbf{x}^{(p)}, \mathbf{u}^{(p)}, t) \\
& \mathbf{h}^{L,(p)} \leq \mathbf{h}^{(p)}(\mathbf{x}^{(p)}, \mathbf{u}^{(p)}, t) \leq \mathbf{h}^{U,(p)} \\
& \mathbf{e}^{L,(p)} \leq \mathbf{e}^{(p)}(\mathbf{x}^{(p)}(t_i^{(p)}), \mathbf{x}^{(p)}(t_f^{(p)}), t_i^{(p)}, t_f^{(p)}) \leq \mathbf{e}^{U,(p)} \\
& \Phi^{L,(p,q)} \leq \Phi^{(p,q)}(\mathbf{x}^{(p)}, \mathbf{x}^{(q)}, \mathbf{u}^{(p)}, \mathbf{u}^{(q)}) \leq \Phi^{U,(p,q)}
\end{aligned} \tag{10}$$

Each phase is discretized with its own set of points, $\{\tau_i^{(p)}\}$ so that,

$$\mathbf{x}^{(p)}(\tau) \approx \hat{\mathbf{x}}^{(p)}(\tau) = \sum_{i=1}^{N} \hat{\mathbf{x}}_i^{(p)} L_i(\tau) \tag{11}$$

so that the full multi-phase NLP is,

$$\begin{aligned}
\min_{\mathbf{u}_i^{(p)}} \quad & \sum_{p=1}^{P} \frac{t_f^{(p)} - t_i^{(p)}}{2} \sum_{k=1}^{N} F^{(p)}(\hat{\mathbf{x}}_k^{(p)}, \hat{\mathbf{u}}_k^{(p)}, \tau_k) \\
\text{s.t.} \quad & \sum_{i=0}^{N} D_{k,i} \hat{\mathbf{x}}_i^{(p)} - \frac{t_f^{(p)} - t_i^{(p)}}{2} \mathbf{f}^{(p)}(\hat{\mathbf{x}}_k^{(p)}, \hat{\mathbf{u}}_k^{(p)}, \tau_k) = \mathbf{0}_n, \quad p = 1, \dots, P, \quad k = 1, \dots, N \\
& \hat{\mathbf{x}}_N^{(p)} - \hat{\mathbf{x}}_0^{(p)} - \frac{t_f^{(p)} - t_i^{(p)}}{2} \sum_{k=1}^{N} \sum_{i=0}^{N} w_k D_{k,i} \hat{\mathbf{x}}_i = \mathbf{0}_n, \quad p = 1, \dots, P \\
& \mathbf{e}^{L,(p)} \leq \mathbf{e}^{(p)}(\hat{\mathbf{x}}_0^{(p)}, \hat{\mathbf{x}}_N^{(p)}, t_i^{(p)}, t_f^{(p)}) \leq \mathbf{e}^{U,(p)}, \quad p = 1, \dots, P \\
& \mathbf{h}^{L,(p)} \leq \mathbf{h}^{(p)}(\hat{\mathbf{x}}_k^{(p)}, \hat{\mathbf{u}}_k^{(p)}, \tau_k) \leq \mathbf{h}^{U,(p)}, \quad k = 1, \dots, N, \quad p = 1, \dots P \\
& \Phi^{L,(p,q)} \leq \Phi^{(p,q)}(\hat{\mathbf{x}}_0^{(p)}, \hat{\mathbf{u}}_0^{(p)}, \hat{\mathbf{x}}_N^{(q)}, \hat{\mathbf{u}}_N^{(q)}) \leq \Phi^{U,(p,q)}, \quad p, q = 1, \dots, P
\end{aligned} \tag{12}$$

To perform the discretization described in this subsection, we use the open-source C++ PSOC package $\mathcal{PSOPT}$ [2].

Next we show that Eq. (12) can be expressed in the typical NLP form [4]. Let $\mathbf{z}^{(p)}$ contain all of the variables

for phase p.

$$\mathbf{z}^{(p)} = \begin{bmatrix} \hat{\mathbf{x}}_0^{(p)} \\ \vdots \\ \hat{\mathbf{x}}_N^{(p)} \\ \hat{\mathbf{u}}_0^{(p)} \\ \vdots \\ \hat{\mathbf{u}}_N^{(p)} \end{bmatrix} \in \mathbb{R}^{(n+m)} \tag{13}$$

Next, let $\mathbf{z}$ contain the variables for every phase,

$$\mathbf{z} = \begin{bmatrix} \mathbf{z}^{(1)} \\ \vdots \\ \mathbf{z}^{(P)} \end{bmatrix} \in \mathbb{R}^{(N+1)(n+m)} \tag{14}$$

With some algebraic manipulation, the entire discretized multi-phase OCP can be rewritten as an NLP in the typical form.

$$\begin{aligned} \min_{\mathbf{z}} \quad & c(\mathbf{z}) \\ \text{s.t.} \quad & \mathbf{g}(\mathbf{z}) = \mathbf{0} \\ & \mathbf{d}(\mathbf{z}) \leq \mathbf{0} \end{aligned} \tag{15}$$

To solve the large-scale NLP in Eq. (15) we employ an interior-point algorithm [4]. Specific details of the algorithm are outside the scope of this paper. We used the open-source C++ package IPOPT [9] to solve each instance of Eq. (15). We direct interested readers who would like to learn more about the technical detailed involved when solving Eq. (15) to the documentation provided with IPOPT.

The optimal solution returned, $\mathbf{z}^*$, is separated into its component parts; first by splitting it into the phases $\mathbf{z}^{(p)*}$, and second by reconstructing the discrete states and control inputs, $\hat{\mathbf{x}}_i^*$ and $\hat{\mathbf{u}}_i^*$. The continuous time control inputs and states are then reconstructed using the Lagrange interpolating polynomials in Eq. (4). With the continuous time states and control inputs, $\mathbf{x}^*(t)$ and $\mathbf{u}^*(t)$, we then verify that the necessary conditions are met to within an acceptable tolerance.

Supplementary Note 9: Necessary Conditions for PSOC Solutions

A closed form solution of Eq. (2) may not exist. Instead one must typically turn to numerical methods, such as PSOC. Nonetheless, it is possible to derive a set of necessary conditions that any solution to Eq. (2) must satisfy based on Pontryagin's minimum principle [3]. Developing these necessary conditions allows one to construct a set of validation criteria with which we may test the quality of any solution returned by the numerical methods. In Ref. [6], the so-called HAMVET procedure has been proposed based on a slightly modified version of Pontryagin's principle to provide the necessary conditions for the general OCP in (2).

The HAMVET procedure is based on the following steps:

- Construction of the Hamiltonian : (H)
- Adjoint equations : (A)
- Minimization of the Hamiltonian : (M)
- Evaluation of the Hamiltonian Value condition : (V)
- Evolution of the Hamiltonian : (E)
- Transversality conditions : (T)

In what follows, we individually describe each one of the steps of the HAMVET procedure. A detailed analysis can be found in Ref. [6].

Construction of the Hamiltonian

The *Hamiltonian* H corresponding to the general OPC problem is

$$H(\boldsymbol{\lambda}, \mathbf{x}, \mathbf{u}, t) = F(\mathbf{x}, \mathbf{u}, t) + \boldsymbol{\lambda}^T \mathbf{f}(\mathbf{x}, \mathbf{u}, t) \tag{16}$$

where $\boldsymbol{\lambda}(t) \in \mathbb{R}^n$ is the *adjoint covector* which is a function of time t. The control input that minimizes the OCP satisfies the Hamiltonian Minimization Condition (HMC), that is,

$$(HMC) \quad \begin{cases} \min\limits_{u(t)} & H(\boldsymbol{\lambda}, \mathbf{x}, \mathbf{u}, t) \\ \text{s.t.} & \mathbf{h}^L \leq \mathbf{h}(\mathbf{x}, \mathbf{u}, t) \leq \mathbf{h}^U \end{cases} \tag{17}$$

Adjoint equations

The Karush-Kuhn-Tucker (KKT) conditions can be used to solve the HMC. We define the *Lagrangian of the Hamiltonian* $\bar{H}$ as

$$\bar{H}(\boldsymbol{\mu}, \boldsymbol{\lambda}, \mathbf{x}, \mathbf{u}, t) = H(\boldsymbol{\lambda}, \mathbf{x}, \mathbf{u}, t) + \boldsymbol{\mu}^T \mathbf{h}(\mathbf{x}, \mathbf{u}, t) \tag{18}$$

where $\boldsymbol{\mu}(t) \in \mathbb{R}^h$ is the *path covector* which is a function of time t. Then the evolution of the adjoint covector $\boldsymbol{\lambda}(t)$ is given by,

$$-\dot{\boldsymbol{\lambda}} = \frac{\partial \bar{H}}{\partial \mathbf{x}} \tag{19}$$

Note that condition in (19) enforces the continuity but not differentiability of $\boldsymbol{\lambda}(t)$. So, the piecewise continuity of $\boldsymbol{\lambda}(t)$ is a necessary condition for an optimal control solution.

Minimization of the Hamiltonian

By the KKT condition, the minimization condition for the Hamiltonian yields

$$\frac{\partial \bar{H}}{\partial \mathbf{u}} = \mathbf{0} \tag{20}$$

with the complementary conditions for path constraints,

$$\begin{cases} \mu_i \leq 0 & if \quad h_i(\mathbf{x}, \mathbf{u}, t) = h_i^L \\ \mu_i = 0 & if \quad h_i^L < h_i(\mathbf{x}, \mathbf{u}, t) < h_i^U \\ \mu_i \geq 0 & if \quad h_i(\mathbf{x}, \mathbf{u}, t) = h_i^U \\ \mu_i \text{ unrestricted} & if \quad h_i^L = h_i^U \end{cases} \tag{21}$$

If there are path constraints, then one of the necessary conditions is

$$\mu_i(t)(h_i - h_i^L)(h_i - h_i^U) = 0 \tag{22}$$

Along with the minimization of the Hamiltonian, there is an endpoint minimization condition (EMC) as well. The endpoint minimization problem is defined as

$$(EMC) \quad \begin{cases} \min & E(\mathbf{x}(t_i), \mathbf{x}(t_f), t_i, t_f) \\ \text{s.t.} & \mathbf{e}^L \leq \mathbf{e}(\mathbf{x}(t_i), \mathbf{x}(t_f), t_i, t_f) \leq \mathbf{e}^U \end{cases} \tag{23}$$

To solve the EMC by KKT, we define the *endpoint Lagrangian* $\bar{E}$ as

$$\begin{aligned} \bar{E}(\boldsymbol{\nu}, \mathbf{x}(t_i), \mathbf{x}(t_f), t_i, t_f) =& E(\mathbf{x}(t_i), \mathbf{x}(t_f), t_i, t_f) \\ &+ \boldsymbol{\nu}^T e(\mathbf{x}(t_i), \mathbf{x}(t_f), t_i, t_f) \end{aligned} \tag{24}$$

where $\boldsymbol{\nu} \in \mathbb{R}^e$ is the *endpoint covector*. Note that, $\boldsymbol{\nu}$ is a constant vector. The complementary conditions for event constraints are given by

$$
\begin{cases}
\nu_i \leq 0 & if \quad e_i(\mathbf{x}(t_i), \mathbf{x}(t_f), t_i, t_f) = e_i^L \\
\nu_i = 0 & if \quad e_i^L < e_i(\mathbf{x}(t_i), \mathbf{x}(t_f), t_i, t_f) < e_i^U \\
\nu_i \geq 0 & if \quad e_i(\mathbf{x}(t_i), \mathbf{x}(t_f), t_i, t_f) = e_i^U \\
\nu_i \text{ unrestricted} & if \quad e_i^L = e_i^U
\end{cases}
\tag{25}
$$

Hamiltonian Value condition

The *lower Hamiltonian* $\mathcal{H}$ is defined as the Hamiltonian evaluated at $\mathbf{u}(t) = \mathbf{u}^*(t)$, the solution to the HMC problem, i.e.,

$$
\mathcal{H} = \min_{\mathbf{u} \in \mathbb{U}} H(\boldsymbol{\lambda}, \mathbf{x}, \mathbf{u}, t) \tag{26}
$$

where $\mathbb{U}$ is the set of feasible control inputs, i.e., they satisfy all of the constraints imposed by Eq. (2). The lower Hamiltonian must satisfy the endpoint value conditions as a regular Hamiltonian

$$
\begin{aligned}
\mathcal{H}(\boldsymbol{\lambda}(t_i), \mathbf{x}(t_i), t_i) &= \frac{\partial \bar{E}}{\partial t_i} \\
\mathcal{H}(\boldsymbol{\lambda}(t_f), \mathbf{x}(t_f), t_f) &= -\frac{\partial \bar{E}}{\partial t_f}
\end{aligned}
\tag{27}
$$

which provides another necessary conditions to check for the optimal control solution.

Time Evolution of the Hamiltonian

As the lower Hamiltonian $\mathcal{H}$ is obtained from the evaluation of the Hamiltonian at the $\mathbf{u}^*(t)$, $\mathbf{x}^*(t)$ and $\boldsymbol{\lambda}^*(t)$, where $\mathbf{x}^*(t)$ and $\boldsymbol{\lambda}^*(t)$ are the states and costates associated with the optimal control solution $\mathbf{u}^*(t)$, $\mathcal{H}$ is a function of time t only. Thus the evolution of the lower Hamiltonian $\mathcal{H}$ can be defined as

$$
\dot{\mathcal{H}} = \frac{d\mathcal{H}}{dt} = \frac{\partial H}{\partial t} \tag{28}
$$

If H in (16) does not depend explicitly on time, then another necessary condition is

$$
\dot{\mathcal{H}} = 0 \quad \text{or} \quad \mathcal{H} = \text{constant} \tag{29}
$$

Transversality conditions

The endpoints of the adjoint covector $\boldsymbol{\lambda}(t)$ are related to the partial derivatives of the endpoint Lagrangian $\bar{E}$. The transversality conditions for the adjoint covector $\boldsymbol{\lambda}(t)$ are

$$\boldsymbol{\lambda}(t_0) = -\frac{\partial \bar{E}}{\partial \mathbf{x}(t_0)} \quad \text{and} \quad \boldsymbol{\lambda}(t_f) = \frac{\partial \bar{E}}{\partial \mathbf{x}_f} \tag{30}$$

Verification of the Necessary Conditions

We have presented the necessary conditions for the general OCP. Next we develop a set of necessary conditions for the solution of the OCP described in the main section. The OCP presented in the main text can be mapped to the general formulation presented in Eq. (2) with the following definitions.

- The state variables $\mathbf{x}(t) = \begin{bmatrix} S(t) & E(t) & A(t) & I_{tp}(t) & I_s(t) & Q(t) & R(t) \end{bmatrix}^T \in \mathbb{R}^7$ and the control input $u(t) \equiv P(t) \in \mathbb{R}$.
- The cost function $J = \int_{t_i}^{t_f} (1 - P(t))dt + \int_{t_i}^{t_f} Q(t)dt$ (see Eq. (7) with $c_p = c_q = 1$ in the main text) so that, from Eq. (2), $E \equiv 0$ and $F = 1 - P(t) + Q(t)$.
- The system dynamics, as presented in Eq. (1), are rewritten here,

$$\dot{\mathbf{x}}(t) = \begin{bmatrix} \dot{S}(t) \\ \dot{E}(t) \\ \dot{A}(t) \\ \dot{I}_{tp}(t) \\ \dot{I}_s(t) \\ \dot{Q}(t) \\ \dot{R}(t) \end{bmatrix} = \mathbf{f}(\mathbf{x}(t), \mathbf{u}(t), t) \tag{31}$$

- The only endpoint constraints are set at the initial time (t_i) and the terminal constraints in Eq. (4) in main text,

$$\mathbf{e}(\mathbf{x}(t_i), \mathbf{x}(t_f), t_i, t_f) = \begin{bmatrix} S(t_i) \\ E(t_i) \\ A(t_i) \\ I_{tp}(t_i) \\ I_s(t_i) \\ Q(t_i) \\ R(t_i) \\ P(t_i) \\ E(t_f) + I_s(t_f) + I_{tp}(t_f) + A(t_f) \end{bmatrix}, \quad \mathbf{e}^L = \begin{bmatrix} \mathbf{x}(t_i) \\ P_2 \\ 0 \end{bmatrix}, \quad \mathbf{e}^U = \begin{bmatrix} \mathbf{x}(t_i) \\ P_2 \\ \epsilon \end{bmatrix} \tag{32}$$

where $\mathbf{x}(t_i) = \begin{bmatrix} S_{t_i} & E_{t_i} & A_{t_i} & I_{tp_{t_i}} & I_{s_{t_i}} & Q_{t_i} & R_{t_i} \end{bmatrix}^T$ are set to the values of the states at t_i.

- Finally, the path constraints consist of the bounds on $I_{tp}(t) + I_s(t)$ and possibly on $P(t)$

$$\mathbf{h}(\mathbf{x}(t), \mathbf{u}(t), t) = \begin{bmatrix} I_{tp}(t) + I_s(t) \\ P(t) \end{bmatrix}, \quad \mathbf{h}^L = \begin{bmatrix} 0 \\ 0 \end{bmatrix}, \quad \mathbf{h}^U = \begin{bmatrix} I_{\max} \\ 1 \end{bmatrix} \tag{33}$$

Developing the necessary conditions allows us to construct a set of validation criteria with which we may test the quality of any solution returned by our numerical methods. For this analysis, we consider the metropolitan city of LA. $I_{\max}$ and ϵ are set to 0.0066 and 10^{-5}, respectively.
Define a vector of time-varying costates (or adjoint variables) as $\boldsymbol{\lambda}(t)$, the Hamiltonian of the OCP in Eq. (2) is defined as,

$$\begin{aligned} H(\boldsymbol{\lambda}, \mathbf{x}, \mathbf{u}, t) &= F(\mathbf{x}, \mathbf{u}, t) + \boldsymbol{\lambda}^T \mathbf{f}(\mathbf{x}, \mathbf{u}, t) \\ &= 1 - P(t) + Q(t) + \boldsymbol{\lambda}^T \mathbf{f}(\mathbf{x}, \mathbf{u}, t) \end{aligned} \tag{34}$$

where $\boldsymbol{\lambda}(t) \in \mathbb{R}^7$ are the costates (or adjoint variables). A solution to Eq. (2) must also be a solution of the following minimization problem.

$$\begin{aligned} \min_{\mathbf{u}(t)} \quad & H(\boldsymbol{\lambda}, \mathbf{x}, \mathbf{u}, t) \\ \text{s.t.} \quad & \mathbf{h}^L \leq \mathbf{h}(\mathbf{x}, \mathbf{u}, t) \leq \mathbf{h}^U \end{aligned} \tag{35}$$

To solve Eq. (35), we define the associated Lagrangian,

$$\begin{aligned} \bar{H}(\boldsymbol{\mu}, \boldsymbol{\lambda}, \mathbf{x}, \mathbf{u}, t) &= H(\boldsymbol{\lambda}, \mathbf{x}, \mathbf{u}, t) + \boldsymbol{\mu}^T \mathbf{h}(\mathbf{x}, \mathbf{u}, t) \\ &= 1 - P(t) + Q(t) + \boldsymbol{\lambda}^T \mathbf{f}(\mathbf{x}, \mathbf{u}, t) + \mu_{I_{tp}+I_s}(I_{tp} + I_s) + \mu_P P(t) \end{aligned} \tag{36}$$

where $\boldsymbol{\mu} = \begin{bmatrix} \mu_{I_{tp}+I_s} & \mu_P \end{bmatrix}^T \in \mathbb{R}^h$ is the copath vector of the path constraints in Eq. (33). A solution to Eq. (35), and thus to our original OCP, must satisfy,

$$\frac{\partial \bar{H}}{\partial P} = \mu_P - 1 + \left(\frac{\partial \mathbf{f}}{\partial P} \right)^T \boldsymbol{\lambda} = 0 \tag{37}$$

where the costates evolve according to the dynamical equation,

$$\dot{\boldsymbol{\lambda}} = -\frac{\partial \bar{H}}{\partial \mathbf{x}} = -\left(\frac{\partial \bar{\mathbf{f}}}{\partial \mathbf{x}} \right)^T \boldsymbol{\lambda} - \begin{bmatrix} \mathbf{0}_3 \\ \mu_{I_{tp}+I_s} \\ \mu_{I_{tp}+I_s} \\ 1 \\ 0 \end{bmatrix} \tag{38}$$

The optimal control input $P^*(t)$, must satisfy the complementarity condition

$$\begin{cases} \mu_P(t) < 0 & \text{if} \quad P^*(t) = 0 \\ \mu_P(t) = 0 & \text{if} \quad 0 < P^*(t) < 1 \\ \mu_P(t) > 0 & \text{if} \quad P^*(t) = 1 \end{cases} \tag{39}$$

The path constraint $I_{tp}(t) + I_s(t)$ must satisfy the another complementary condition

$$\begin{cases} \mu_{I_{tp}+I_s} < 0 & \text{if} \quad I_{tp}(t) + I_s(t) = 0 \\ \mu_{I_{tp}+I_s} = 0 & \text{if} \quad 0 < I_{tp}(t) + I_s(t) < I_{\max} \\ \mu_{I_{tp}+I_s} > 0 & \text{if} \quad I_{tp}(t) + I_s(t) = I_{\max} \end{cases} \tag{40}$$

In Fig. 23(A) and (B), we plot $P^*(t)$ and $\mu_P(t)$, respectively. We note that $0 < P^*(t) < 1$ and $\mu_P(t)$ remains at zero over the the control time interval. In Fig. 23(C) and (D), we plot $I_{tp}(t) + I_s(t)$ and $\mu_{I_{tp}+I_s}(t)$, respectively. Note that $\mu_{I_{tp}+I_s}(t) > 0$ when $I_{tp}(t) + I_s(t) = I_{\max}$, $\mu_{I_{tp}+I_s}(t) = 0$ when $0 < I_{tp}(t) + I_s(t) < I_{\max}$, and when $\mu_{I_{tp}+I_s}(t) < 0$ when $I_{tp}(t)+I_s(t) \approx 0$. These necessary conditions are obtained from the complementary conditions while minimizing the Hamiltonian.
Similarly, in order to minimize the endpoints constraints, the endpoint Lagrangian $\bar{E}$ in Eq. (24) becomes,

$$\bar{E} = \boldsymbol{\nu}^T \mathbf{e}(\mathbf{x}(t_0), \mathbf{x}(t_f), t_0, t_f) \tag{41}$$

The endpoint covector must satisfy the conditions in Eq. (25), but it is reasonable to verify at least ν_9 associated with the terminal constraint $E_{tf} + I_s(t_f) + I_{tp}(t_f) + A(t_f)$. We obtained $\nu_9 < 0$ suggesting that $E_{tf} + I_s(t_f) + I_{tp}(t_f) + A(t_f) \approx 0$.
Let us now assume that we have solved Eq. (35), that is,

$$\mathcal{H}(t) = \min_{P(t)} H(\boldsymbol{\lambda}, \mathbf{x}, P^*, t) \tag{42}$$

The evolution of the Hamiltonian at the optimal solution can be written,

$$\frac{d\mathcal{H}}{dt} = \frac{\partial H}{\partial t} \tag{43}$$

where, since in our OCP, H does not explicitly depend on time, we expect that $d\mathcal{H}/dt = 0$ and so $\mathcal{H}$ should be constant. This is the next validation condition. In Fig. 23(E), we plot $\mathcal{H}(t)$ and it is clear that $\mathcal{H}(t)$ is constant over $t_i < t < t_f$. The transversality conditions in Eq. (30) are also verified from the numerical solution of $\boldsymbol{\lambda}(t)$. In Fig. 23(F), we plot the relative discretization error at each time t. From Fig. 23, we conclude that the necessary condition derived for the optimal control input are satisfied.

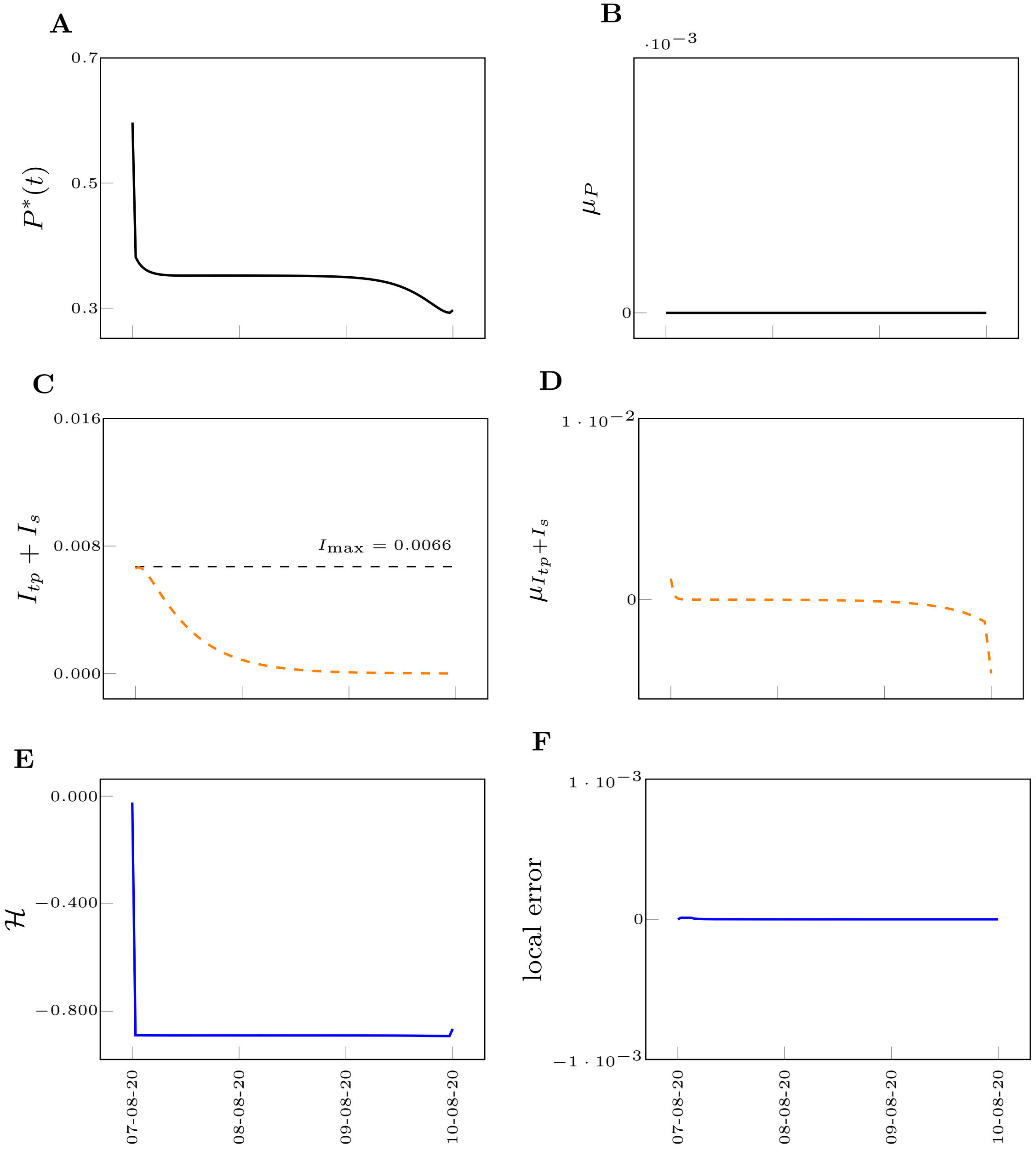

Figure 23: HAMVET procedure applied to our solution of the optimal control problem for the metropolitan city of LA. $I_{\max}$ and ϵ are set to 0.0066 and 10^{-5}, respectively. A) The optimal time evolution of the control input $P^*(t)$. B) The time evolution of the path covector μ_P. C) The time evolution of $I_{tp}(t) + I_s(t)$. D) The time evolution of the path covector $\mu_{I_{tp}+I_s}(t)$. E) The time evolution of the lower Hamiltonian $\mathcal{H}$. F) The relative local discretization error at each time t.

Supplementary Note 10: Sensitivity Analysis

It is important to check the sensitivity of the optimal control solution to small perturbations of the system parameters. To this aim, we perturb the parameter β, i.e., the most important parameter of the model, and compute how much the optimal control input changes. The other parameters remain unchanged. We define ξ as the average normalized variation in the optimal control input when β is varied by a quantity equal to $\delta\%$,

$$\xi = \left\langle \frac{|P^*(t) - P^*_\delta(t)|}{P^*(t)} \right\rangle \tag{44}$$

where $\langle . \rangle$ indicates a time average over the control time, $P^*(t)$ is the optimal input for the original value of β and $P^*_\delta(t)$ is the optimal control input for $(1+\delta\%)\beta$. For this analysis, we consider the metropolitan city of Seattle. $I_{\max}$ and ϵ are set to 0.0046 and 10^{-5}, respectively. In Fig. 24, we plot ξ vs. $\delta\%$.

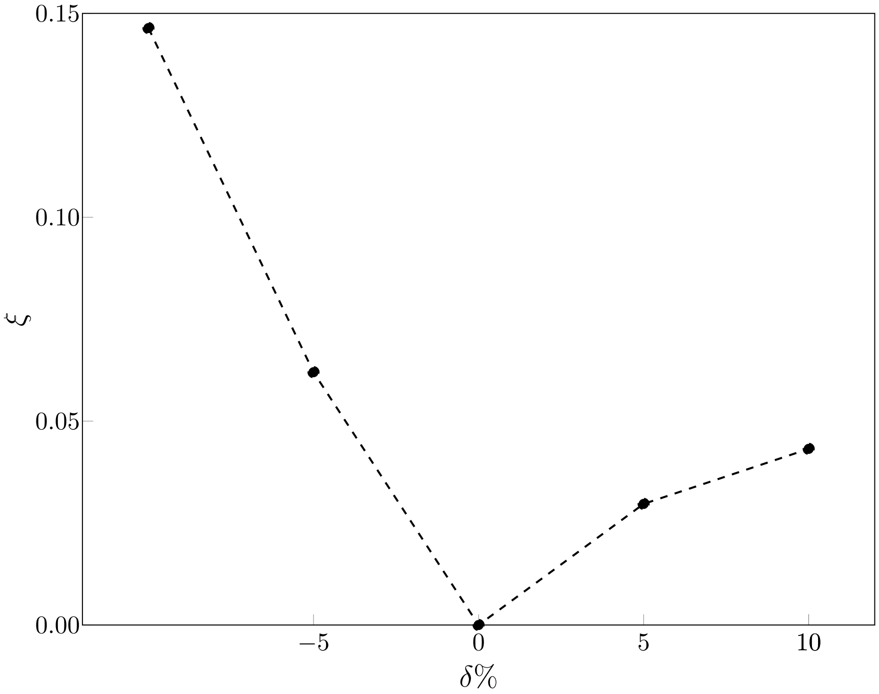

Figure 24: Sensitivity analysis of the optimal control input to a perturbation in the parameter β for the metropolitan city of Seattle. The average variation ξ in the optimal control input vs. the change δ in the parameter β. $I_{\max}$ and ϵ are set to 0.0046 and 10^{-5}, respectively.

Supplementary Note 11: Incorporation of Vaccinations in the Model and Effects on the Optimal Control Solutions

Figure 25 shows very good agreement between the daily new case counts reported by local health administrations for the city of Seattle over the extended period from January 21, 2020 to December 14, 2020 (blue symbols) and the daily new cases obtained by integrating Eq. (2) of the main text after parametrization of a 4-phase model (four social-distancing episodes with piece wise-linear transition of $P(t)$; red solid line).

According to [1] a total of 1.36 million doses have been administered in the US on February 1 2021, with the number of doses per day increasing roughly linearly with time. Thus we model the number of doses administered per day as $\kappa \times (t - t_v)$, where we set the time t_v to be Dec-14-2020, the date in which vaccinations began in the US. It becomes important to estimate the linear rate of increase κ. There is a total of 49 days from Dec-14-2020 to Feb-1-2021. Assuming each person gets 2 vaccine doses, we have $\kappa \times 49 = 1.36M/2$ leading to $\kappa = 13878$ people/day nationally. The MSA of Seattle has population of roughly $4M$, which is about 1.29% of the total US population. This means the κ for Seattle should be equal to roughly $13878 \times 0.0129 = 180$ (person/day.) In the non-dimensional case, this corresponds to $\kappa = 0.000045$ (1/day.)

Figure 26 shows the state time evolutions corresponding to the optimal control inputs shown in Figure 7 of the main text. The left plots are for a shorter control horizon, $T_{cont} = (t_f - t_i) = 130$ days, while the right plots are for a longer control horizon, $T_{cont} = (t_f - t_i) = 150$ days. We compare the two cases that $\kappa = 0$, i.e., we do not model the effects of vaccinations (in A and B), and that $\kappa = 0.000045$, i.e., we model the effects of vaccinations (in C and D.)

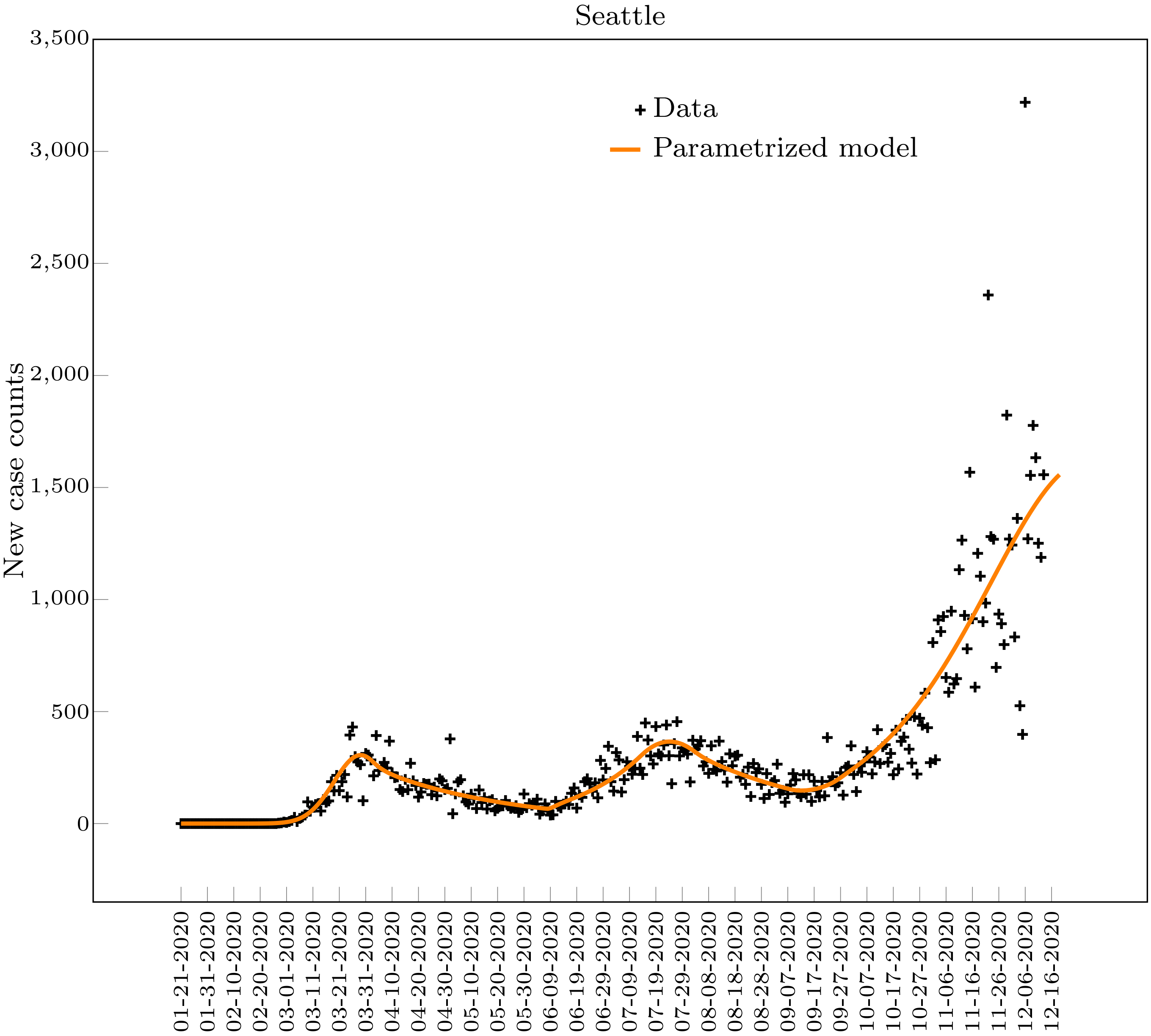

Figure 25: New case counts from January 21, 2020 to December 14, 2020 for the Metropolitan Statistical Areas of Seattle. Blue symbols are daily new case counts reported by the local health administration. The solid line is the daily new cases obtained by integrating Eq. (2) after parametrization of the model.

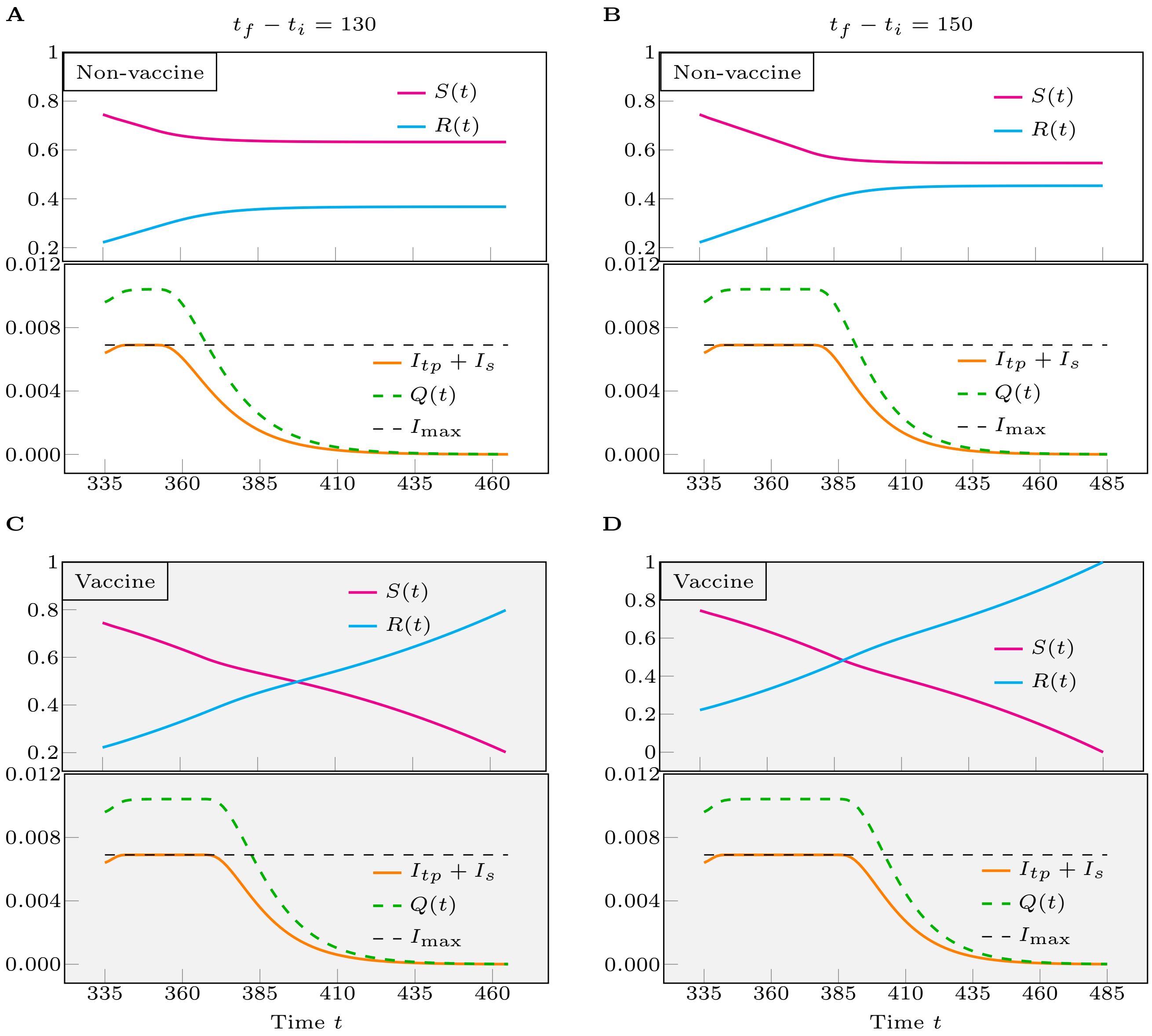

Figure 26: Time evolution of the states subject to the optimal control inputs shown in Fig. 7 of the main text for the city of Seattle. A and B are for the case that vaccinations are not modeled ($\kappa = 0$), while C and D are for the case that vaccinations are modeled ($\kappa = 0.000045$). Plots on the left are for a shorter control horizon $(t_f - t_i) = 130$ days while the plots on the right are for a longer control horizon $(t_f - t_i) = 150$ days. $I_{\max}$ are chosen as the maximum of the range in Table 3 ($\rho = 1$) and $c_p = c_q = 1$.

Supplementary Note 12: Non-Normalized Equations

Consider the non-normalized quantities in the variables $\hat{S}(t) = NS(t)$, $\hat{E}(t) = NE(t)$, $\hat{A}(t) = NA(t)$, $\hat{I}(t) = NI(t)$, $\hat{I}_{tp}(t) = NI_{tp}(t)$, $\hat{Q}(t) = NQ(t)$, $\hat{R}(t) = NR(t)$, where N is the total number of individuals in the population, such as $\hat{S}(t) + \hat{E}(t) + \hat{A}(t) + \hat{I}(t) + +\hat{I}_{tp}(t) + \hat{Q}(t) + \hat{R}(t) = N$ at any time. The non-normalized quantities evolve based to following equations,

$$\dot{\hat{S}}(t) = -\hat{\beta} P^2(t)\,\hat{S}(t)\left[\hat{I}(t) + \hat{I}_{tp}(t) + \mu\hat{A}(t)\right] \tag{45a}$$

$$\dot{\hat{E}}(t) = \hat{\beta} P^2(t)\,\hat{S}(t)\left[\hat{I}(t) + \hat{I}_{sq}(t) + \hat{I}_{tp}(t) + \mu\hat{A}(t)\right] - \lambda\hat{E}(t) \tag{45b}$$

$$\dot{\hat{A}}(t) = \lambda(1-\sigma)\,\hat{E}(t) - \gamma_A\hat{A}(t) \tag{45c}$$

$$\dot{\hat{I}}_{tp}(t) = p_{test}\lambda\sigma\hat{E}(t) - [\gamma_I + \gamma_{tp}]\,\hat{I}_{tp}(t) \tag{45d}$$

$$\dot{\hat{I}}(t) = (1 - p_{sq} - p_{test})\,\lambda\sigma\hat{E}(t) - \gamma_I\hat{I}(t) \tag{45e}$$

$$\dot{\hat{Q}}(t) = \gamma_{tp}\hat{I}_{tp}(t) + p_{sq}\lambda\sigma\hat{E}(t) - \gamma_I\hat{Q}(t) \tag{45f}$$

$$\dot{\hat{R}}(t) = \gamma_A\hat{A}(t) + \gamma_I\left[\hat{I}(t) + \hat{I}_{tp}(t) + \hat{Q}(t)\right]. \tag{45g}$$

where $\hat{\beta} = N^{-1}\beta$. All other parameters are the same as defined in Eq. 2 of the main manuscript.

Supplementary Note 13: Model incorporating limited testing

An extended version of the model that incorporates limited testing is the following.

$$\dot{S}(t) = -\beta P^2(t)\,S(t)\,[I(t) + I_{tp}(t) + \mu A(t)] \tag{46a}$$

$$\dot{E}(t) = \beta P^2(t)\,S(t)\,[I(t) + I_{tp}(t) + \mu A(t)] - \lambda E(t) \tag{46b}$$

$$\dot{A}(t) = \lambda(1-\sigma)\,E(t) - \gamma_A A(t) \tag{46c}$$

$$\dot{I}_{sq}(t) = p_{sq}\lambda\sigma E(t) - [\gamma_I + \gamma_{sq}]\,I_{sq}(t) \tag{46d}$$

$$\dot{I}_{tp}(t) = T\frac{p_{test}\sigma\lambda E}{p_{test}\sigma\lambda E + D} \wedge p_{test}\sigma\lambda E - (\gamma_I + \gamma_{tp})\,I_{tp}(t) \tag{46e}$$

$$\dot{I}(t) = (1 - p_{sq})\,\lambda\sigma E(t) - T\frac{p_{test}\sigma\lambda E}{p_{test}\sigma\lambda E + D} \wedge \sigma p_{test}\lambda E - \gamma_I I(t) \tag{46f}$$

$$\dot{Q}(t) = \gamma_{tp}I_{tp}(t) + p_{sq}\lambda\sigma E - \gamma_I Q(t) \tag{46g}$$

$$\dot{R}(t) = \gamma_A A(t) + \gamma_I\,[I(t) + I_{tp}(t) + Q(t)]. \tag{46h}$$

The flux of population that will be tested is $p_{test}\sigma\lambda E$, and the positive detection probability is

$$\frac{p_{test}\sigma\lambda E}{p_{test}\sigma\lambda E + D}. \tag{47}$$

This is to assume that the number of testing kits is less than the total people needed to be tested (scarcity) which can be described as: number of people which require testing $\approx (p_{test}\sigma\lambda E + D)\,\Delta t$ in a small $\Delta t \ll 1$ time. Otherwise, we will assume a probability of 1 of detecting all those with COVID-19 while ignoring the false negatives. The variable T is the flux of testing kits which are generated (so unit is number per day). We use the standard notation $a \wedge b = \min\{a, b\}$. The testing flux T is most-likely lower than $p_{test}\lambda E$, but if we take a temporally averaged value of T, it is likely that we can get everyone a kit at the early stage. The above general model considers the possibility that testing kits are scarce (i.e., $T < (p_{test}\sigma\lambda E + D)$). However, for NY, at this moment (subdued pandemic), it is likely that the resource is not scarce.

References

[1] "Daily COVID-19 vaccine doses administered". Our World in data. https://ourworldindata.org/grapher/cumulative-covid-vaccinations. February 2020.

[2] Victor M Becerra. Solving complex optimal control problems at no cost with psopt. In *2010 IEEE International Symposium on Computer-Aided Control System Design*, pages 1391–1396. IEEE, 2010.

[3] Donald E Kirk. *Optimal control theory: an introduction.* Courier Corporation, 2004.

[4] Jorge Nocedal and Stephen Wright. *Numerical optimization.* Springer Science & Business Media, 2006.

[5] Anil V Rao. A survey of numerical methods for optimal control. *Advances in the Astronautical Sciences*, 135(1):497–528, 2009.

[6] I Michael Ross. *A primer on Pontryagin's principle in optimal control.* Collegiate publishers, 2015.

[7] I Michael Ross and Mark Karpenko. A review of pseudospectral optimal control: From theory to flight. *Annual Reviews in Control*, 36(2):182–197, 2012.

[8] Nick Warren Ruktanonchai, JR Floyd, Shengjie Lai, Corrine Warren Ruktanonchai, Adam Sadilek, Pedro Rente-Lourenco, Xue Ben, Alessandra Carioli, Joshua Gwinn, JE Steele, et al. Assessing the impact of coordinated covid-19 exit strategies across europe. *Science*, 2020.

[9] Andreas Wächter and Lorenz T Biegler. On the implementation of an interior-point filter line-search algorithm for large-scale nonlinear programming. *Mathematical programming*, 106(1):25–57, 2006.
